# The Experience of Implementing a National Antimicrobial Resistance Surveillance System in Brazil

**DOI:** 10.3389/fpubh.2020.575536

**Published:** 2021-01-14

**Authors:** Marcelo Pillonetto, Regiane Tigulini de Souza Jordão, Gabriel Savogin Andraus, Ricardo Bergamo, Fabiano Barreto Rocha, Mayara Caroline Onishi, Bernardo Montesanti Machado de Almeida, Keite da Silva Nogueira, Amanda Dal Lin, Viviane Maria de Carvalho Hessel Dias, André Luiz de Abreu

**Affiliations:** ^1^State Laboratory for Public Health, Department of Health Assistance and Surveillance, Secretary of Health, Curitiba, Brazil; ^2^Laboratory of Medical Microbiology, School of Medicine, Pontifical Catholic University, Curitiba, Brazil; ^3^General Coordination for Public Health Laboratories, Health Surveillance Secretary, Ministry of Health, Brasília, Brazil; ^4^Infection Control Team and Clinical Microbiology Laboratory, Hospital Nossa Senhora das Graças, Curitiba, Brazil; ^5^Infection Control Team and Clinical Microbiology Laboratory, Hospital de Clínicas, Federal University of Parana, Curitiba, Brazil; ^6^Infection Control Team and Clinical Microbiology Laboratory, Hospital Marcelino Champagnat, Marista Group, Curitiba, Brazil

**Keywords:** surveillance, antimicrobial resistance, multiresistant bacteria, antimicrobial susceptibility tests, public health, GLASS - Global Antimicrobial Resistance Surveillance System, BR-GLASS, Brazil

## Abstract

Antimicrobial resistance (AMR) is a major public health threat of global proportions, which has the potential to lead to approximately ten million deaths per year by 2050. Pressured by this wicked problem, in 2014, the World Health Organization launched a call for member states to share AMR data through the implementation of the Global Antimicrobial Resistance Surveillance System (GLASS), to appropriately scale and monitor the general situation world-widely. In 2017, Brazil joined GLASS and, in 2018, started its own national antimicrobial surveillance program (BR-GLASS) to understand the impact of resistance in the country. We compiled data obtained from the complete routine of three hospitals' microbiology labs during the year of 2018. This pilot data sums up to 200,874 antimicrobial susceptibility test results from 11,347 isolates. It represents 119 different microorganisms recovered from 44 distinct types of clinical samples. Specimens came from patients originating from 301 Brazilian cities, with 4,950 of these isolates from presumed Healthcare-Associated Infections (HAIs) and the other 6,397 community-acquired cases. The female population offered 58% of the collected samples, while the other 42% were of male origin. The urinary tract was the most common topography (6,372/11,347 isolates), followed by blood samples (2,072/11,347). Gram-negative predominated the bacterial isolates: *Escherichia coli* was the most prevalent in general, representing 4,030 isolates (89.0% of these from the urinary tract). Coagulase-negative *Staphylococci* were the most prevalent bacteria in blood samples. Besides these two species, the ESKAPE group have consolidated their prevalence. Regarding drug susceptibility results, 141,648 (70.5%) were susceptible, 9,950 (4.9%) intermediate, and 49,276 (24.5%) resistant. *Acinetobacter baumannii* was the most worrisome microorganism, with 65.3% of the overall antimicrobial susceptibility tests showing resistance, followed by ESBL-producing *Klebsiella pneumoniae*, with a global resistance rate of 59%. Although this is a pilot project (still limited to one state), this database shows the importance of a nation-wide surveillance program,[153mm][-12mm] Q14 especially considering it already had patients coming from 301 distinct counties and 18 different states. The BR-GLASS Program is an ongoing project that intends to encompass at least 95 hospitals distributed in all five geographical regions in Brazil within the next 5 years.

## Introduction

Antimicrobial resistance (AMR) has become a significant global public health threat, which could lead to up to ten million deaths per year by 2050 (1). As the effectiveness of our drug arsenal was progressively shortened by the dissemination and development of resistance genes amongst both the nosocomial and community-borne bacterial populations, the World Health Organization (WHO) launched the Global Action Plan (GAP) on Antimicrobial Resistance in 2015 to fight AMR. Global Action Plan proposed to initiate GLASS–the Global Antimicrobial Resistance Surveillance System, an AMR surveillance system, which combines inpatient, outpatient, laboratory, and epidemiological surveillance data to enhance understanding of the impact AMR poses on healthcare and society (2). Accordingly, adequate surveillance is a recurrent subject in the WHO Global Action Plan, and, as such, it is strongly recommended that countries implement or improve their National Action Plan (NAP), including a Surveillance Program, to assess local resistance patterns and trends appropriately.

Brazil has a long-time Surveillance Program lead by the National Health Regulatory Agency (ANVISA) that compiles data from Healthcare-Associated Infections (HAIs). On the other hand, the GLASS Program proposes a different approach to data collection and analysis: a more comprehensive one health nature that includes resistance information of isolates from both inpatients and outpatients. Also, it can return real-time feedback for health services involved, through dashboards. Since 2014, ANVISA collects and interprets systematic information from all hospitals with ICU beds regarding HAIs, especially concerning catheter-related bloodstream infection (CR-BSI) and their microbial resistance markers. In 2017, 72% of Brazilian hospitals participated, offering laboratory-confirmed bloodstream infection information. In adult Intensive Care Units (ICU), such data showed high resistance rates with, firstly, carbapenem-resistant *Acinetobacter* spp (77%), followed by oxacillin-resistant coagulase-negative *Staphylococci* (CoNS) (72%). In Pediatric ICUs, the high rates continued, but the primary pathogens were inverted, with the percentage of oxacillin-resistant CoNS at 73.4% and carbapenem-resistant *Acinetobacter* spp at 48.6%. The Neonatal ICUs (NICU) showed equally high resistance rates, with 78.4% for oxacillin-resistant CoNS, and 43.5% for carbapenem-resistant *Pseudomonas aeruginosa* (3).

Despite the outstanding surveillance of HAIs by ANVISA, there persisted a gap of information in Brazil about AMR, especially considering the One Health approach, which establishes the need to monitor not only resistant nosocomial bacteria but also the more common community-borne isolates.

Between 2016 and 2017, national discussions took place to prepare the Brazilian NAP on AMR. One of its objectives included joining GLASS. Also, we plan to enlarge the scope under surveillance significantly. This task was accomplished by analyzing all possible samples and etiologic agents in health care settings, still under the same principles and methodologies of GLASS. In 2017 Brazil joined GLASS and started its National Surveillance Program on Antimicrobial Resistance (BR-GLASS). It began with its pilot in 2018 in the state of Parana—Southern Brazil. Considering that in Brazil there are 4,324 clinical laboratories and 6,678 hospitals (CNES, 2017–http://cnes.datasus.gov.br/), such a pilot study allowed the creation of an information system that enables to work with qualified and validated data. These observations came from the vast body of data points created by the routine of microbiology laboratories. This system consists of a web server submission tool for collecting clinical and microbiological data from reporting sentinels. It is associated with a platform built from Elastic Search as an open-source data analysis tool and Kibana as its plugin (https://www.elastic.co). It allows us to visualize compiled BR-GLASS data in the form of interactive charts and tables (dashboards). In July 2019, Brazil reported its first consolidated data from BR-GLASS to GLASS, after qualifying and validating it. With a total of three hospitals submitting their data to the system, so far, we were able to analyze 11,347 isolates within BR–GLASS and reported 3,558 to WHO–GLASS.

## Materials and Methods

### GLASS Program Plan

After deciding to participate in GLASS in December 2017, the Brazilian Ministry of Health designated CGLAB (The General Coordination of Public Health Laboratories) at the Department Of Strategic Actions in Health Surveillance (DAEVS) as the National Coordination Center for BR-GLASS and also determined the Central Public Health Laboratory of Parana (LACEN-PR) as the National Reference Laboratory (NRL) responsible for starting the BR-GLASS Program. For thirteen months (December 2017—December 2018), a multi-professional team of microbiologists, infectious disease control personnel, Information Technology specialists, and Statistics experts elaborated a strategic plan for initiating the National Surveillance Program on Antimicrobial Resistance (BR-GLASS). As Brazil is a continental country with more than 210 million inhabitants distributed throughout 26 states and one federal district (http://www.ibge.gov.br), the team decided to begin the program with a pilot project to be implemented in at least three hospitals in one state, during the first year. The State of Parana was chosen to house the pilot due to its technical capability and epidemiological factors, such as previous experience in controlling HAIs, public health laboratory expertise, extensive health surveillance, and ample dynamic hospital settings (see Supplementary Figure 1). Geographically, Parana is located in southern Brazil, with more than 11.46 million inhabitants distributed in an area of 199.315 km^2^ (http://www.ibge.gov.br), conferring it a population close to that of relevant countries such as Belgium, Greece, and Bolivia. Its territorial extension is similar to Senegal and larger than Greece and South Korea. Its population density is 56.9 hab/km^2^, comparable to Panama, Georgia, and Nicaragua, but superior to South Africa and Colombia. Parana has 429 hospitals, which have 28,340 beds and were attended in 2007 by 40,187 physicians. Additionally, Parana presents circa 24.1 hospital beds per 10,000 inhabitants, a metric similar to that of Sweden, Turkey, Denmark, Ireland, Georgia, and the United Kingdom (4).

The original BR-GLASS plan establishes that the program will expand gradually to other states every year, reaching 95 sentinel hospitals in at least 15 states from the five different geographical regions in Brazil at the end of 5 years.

### Data Submission and BR-GLASS Analysis

BR-GLASS IT team received data from Hospital Information Systems (HIS) as a compiled table. It contained all necessary information about the patient and the isolate(s), such as hospital registration number, age, gender, patient origin (hospital or community), date of the internship, time of sample collection, biological sample, bacterial identification, antimicrobial susceptibility test results and so on. For details, see Supplementary Figure 2.

**Figure 1:**
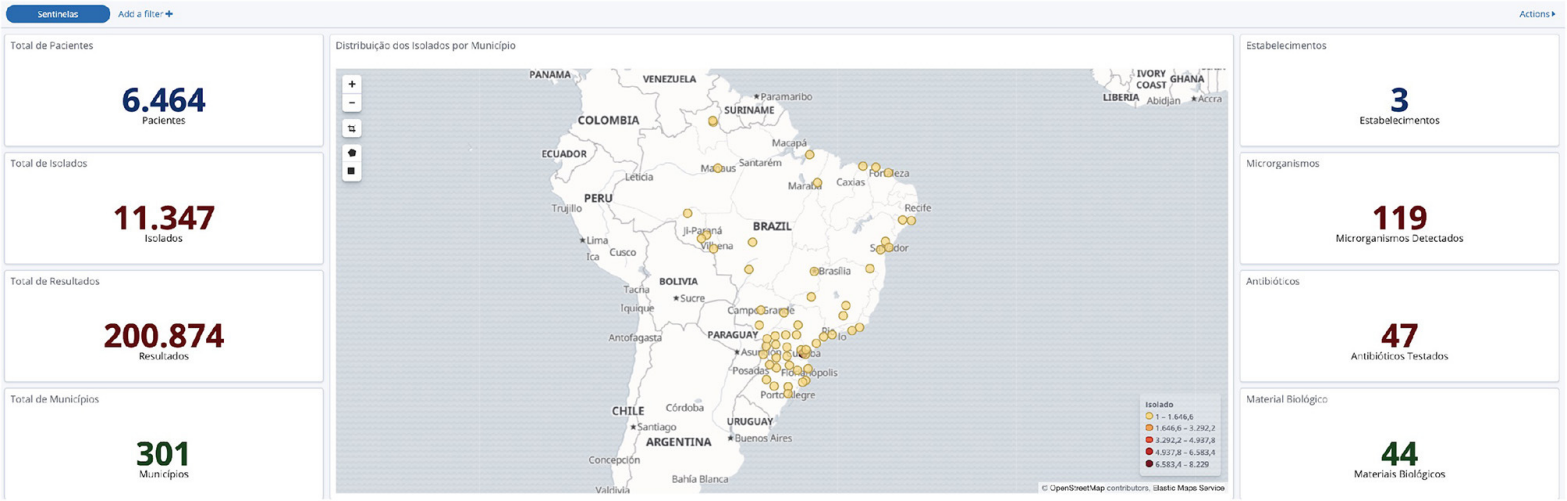
General data obtained from the BR-GLASS Platform.

**Figure 2:**
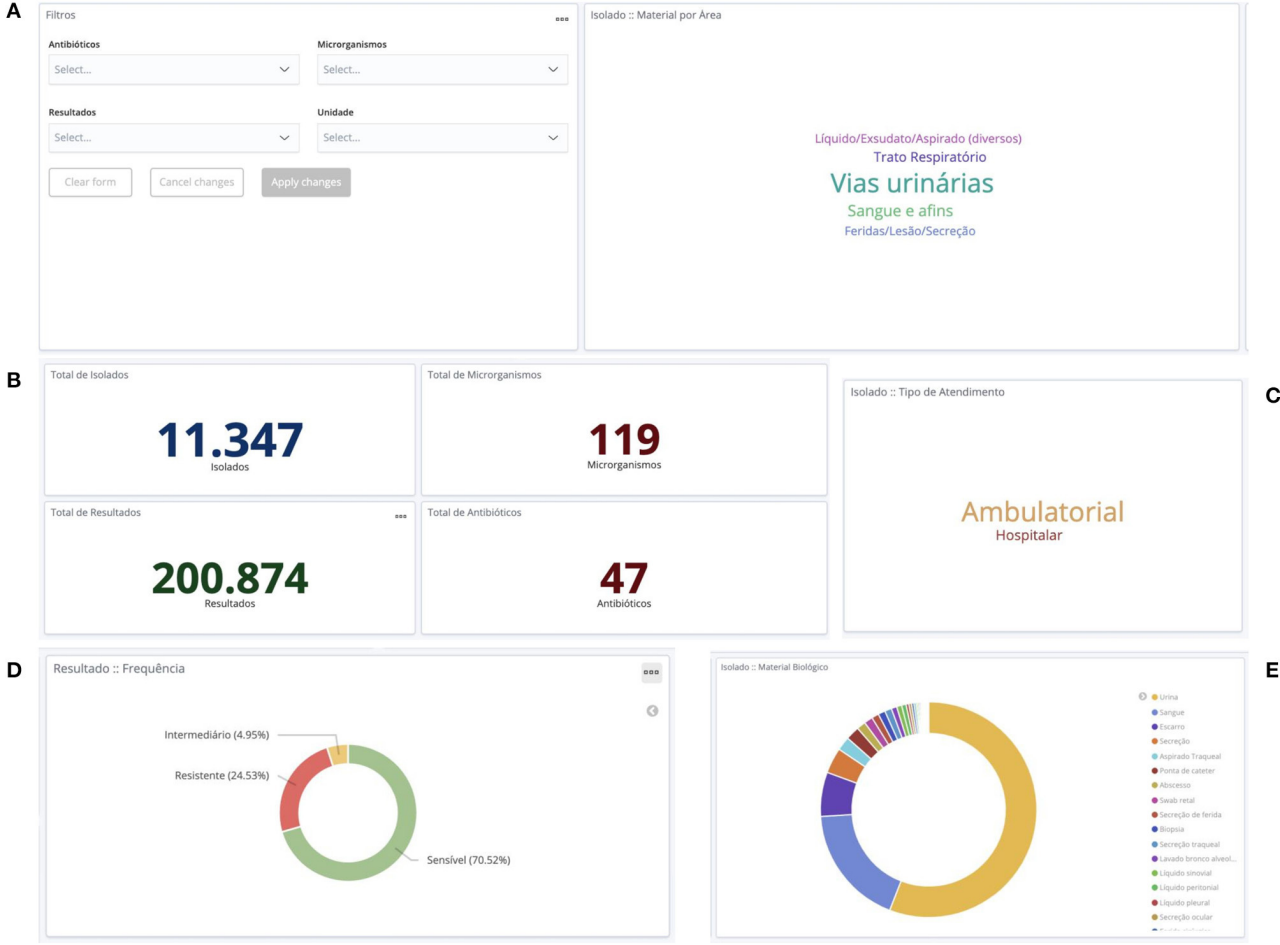
Opening dashboards of the BR-GLASS platform. **(A)** Left: System's Filters for antibiotics, microorganisms, results (susceptible, intermediate or resistant) and Ward; Right: Word Cloud for isolates by anatomical site. **(B)** Total isolates (year 2018), different species, total results and total kind of antibiotics tested. **(C)** Word Cloud for outpatients (Ambulatorial) and inpatients (Hospitalar). **(D)** Total frequency of susceptible, resistant and intermediate isolates. **(E)** Percentage of samples by sample origin.

**Figure 3:**
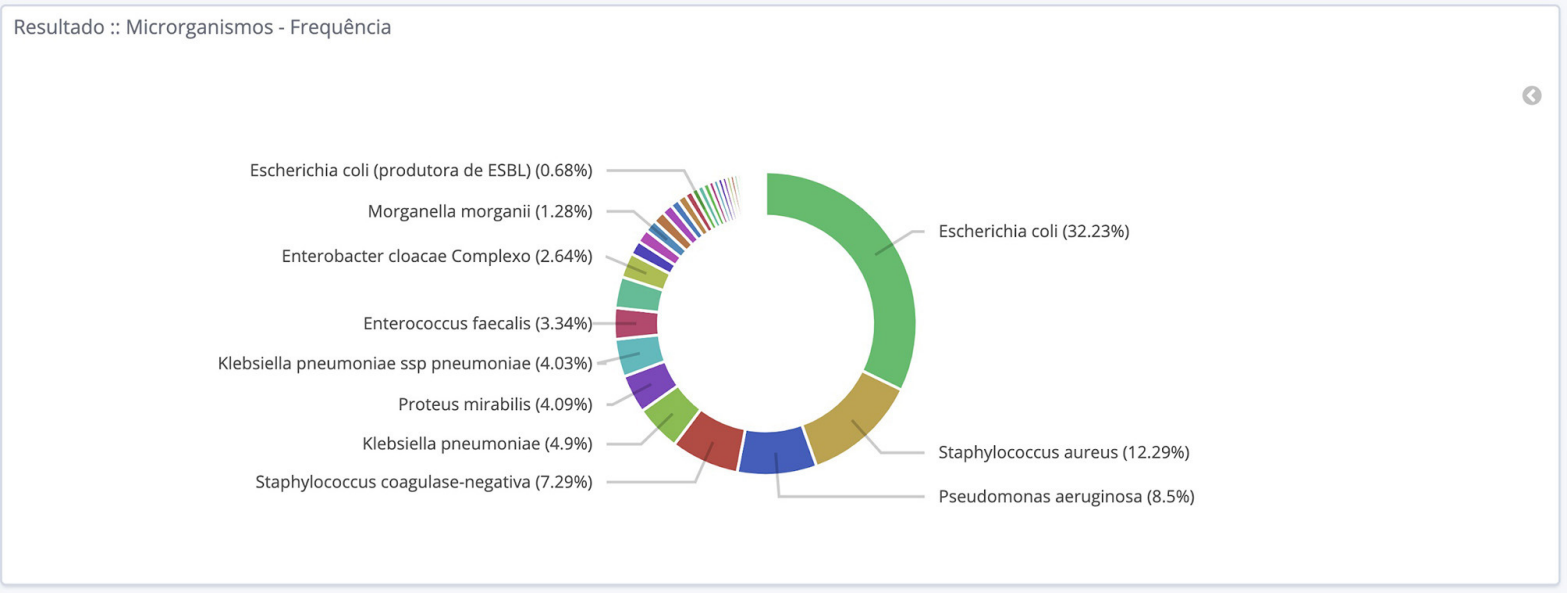
Total frequency of microorganism isolated during the year 2018 at hospitals participating in the BR-GLASS Program.

**Figure 4:**
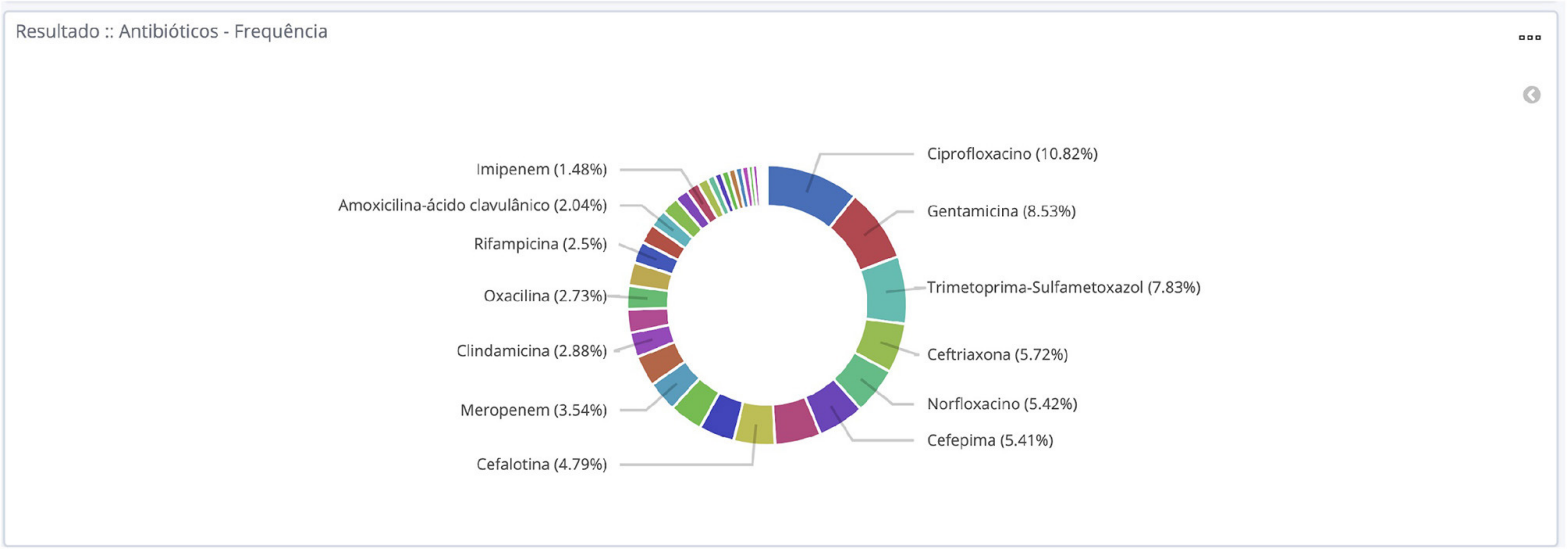
Total frequency of antimicrobials tested during the year 2018 at hospitals participating in the BR-GLASS Program.

**Figure 5:**
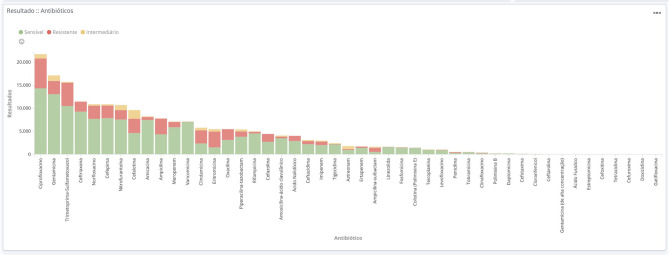
Absolute frequency of results from antimicrobials tested during the year 2018 at hospitals participating in the BR-GLASS Program.

Once our IT team received the data, it went through systematic quality checks to avoid incomplete information, isolates duplication, and unnecessary drug results for a specific pathogen. After data compilation, a series of dashboards were created with the use of an Elastic Co. tool known as Kibana (https://www.elastic.co/guide/en/kibana/current/dashboard.html). This tool allows the real-time visualization of aggregated data as graphs, maps, word clouds, and tables (see Figures 1–5 and Supplementary Figures 3a-e).

The hospital team (ICP, microbiologists, IT specialist) in each sentinel may consult a webpage (https://gae.saude.gov.br/) that allows them to visualize and analyze the Hospital data where he/or she works. Access is made only after authentication via a specific login and password. Only the National Coordinator Center and the National Reference Laboratory are allowed to access the combined data from all the Hospitals. Nonetheless, the program will publish the overall results annually, so all public health professionals (MDs, infection control practitioners, microbiologists, and hospital managers, will have access to the compiled data.

One of the main advantages of this tool is that it allows for a simple and intuitive means of filtering and analyzing data online almost instantaneously by just selecting on the screen the bacterial species, the type of sample or the antibiotic one desires to have more information (see Supplementary Figures 3d,e).

All samples submitted to the routine microbiology laboratory were eligible for submission to the program, although we included only positive cultures with antimicrobial susceptibility test results. All three institutions used automated methods to determine the Minimum Inhibitory Concentration (MIC) for the tested antimicrobials, except when this method was not recommended (i.e., vancomycin for *Staphylococcus aureus*) Infection was considered community-acquired if the sample was collected in outpatients or collected in <48 h after admission. Exclusion criteria were more than one isolate of the same species from the same sample within twelve months; incomplete results; no antimicrobial susceptibility test results available.

### Comparative Analysis

For this study, all positive cultures submitted to the microbiology lab were included, not only resistant strains. Each drug was analyzed for its susceptibility data, pooled against all microorganisms. Subsequently, the main bacterial species were analyzed against the most important group of drugs routinely tested for each species. Due to the large amount of species present in our database we decide to discuss only the most relevant pathogens for Hospital-Associated Infection scenario—The ESKAPE group, as defined by Rice (5) which originally includes *Enterobacter* spp*, S. aureus, Klebsiella pneumoniae, Acinetobacter baumannii, P. aeruginosa*, and *Enterococcus faecium*. Due to limitations of data for comparative analysis we did not included *Enterobacter* spp in the discussion. Instead, we analyzed the resistance of *Escherichia coli*, because of its increasing resistance patterns. Although *E. faecalis* is more prevalent, here we will discuss *E. faecium* due to its broader resistance profile. The community-acquired isolates will be discussed in a future publication.

To compare our results to other countries, we used data from the 2018 EARS-Net Report (6), 2019 CAESAR Report (7), 2014 ReLAVRA Report (8), and 2017 Kor-GLASS Report (9). Such reports were chosen due to the similarity of the data presented to ours (see Supplementary Table 1). Using such data, when appropriate, international average resistance rates were calculated, being referred to in the discussion as the calculated average. It is important to note that the EARS-NET report refers to data pertaining to 2018, the CAESAR report to 2018, the ReLAVRA report to 2013, and the Kor-GLASS report to 2016.

In order to compare 2018 BR-GLASS data to AMR data from other countries we estimated 95% confidence intervals for our resistance rates. AMR data was considered comparable to BR-GLASS if the country's point estimation for resistance was contemplated within the 95% confidence interval estimation for our resistance rates.

## Results

From a total of 429 hospitals in Parana, 32 met the cut-off criteria of having more than 20 ICU beds in general (adult ICUs plus NICUs plus other ICU modalities). These 32 institutions were invited by the Brazilian Ministry of Health to participate voluntarily in BR-GLASS, via e-mail and written letter. The invited health services took an online survey (FORMSUS—see Supplementary Figure 1) that measured its capability to take part in the program as a Surveillance Site. The requirements to participate in the program were: more than 20 ICU beds, both inpatient and outpatient services, an active infection control team, an external quality control program at the laboratory. Desirable features were at least one hospital epidemiologist, one clinical microbiologist, execution of phenotypic and genotypic resistance gene detection protocols, participation as a Surveillance site for the National Health Surveillance Agency–ANVISA Program, and a minimal score (20 points, according to the questions shown on Supplementary Figure 1). Eleven out of 32 hospitals (34.4%) took the survey. All eleven responding hospitals fulfilled the minimum requirements and score and were enrolled in the program. In December 2018, the program started, and by October 2019, three hospitals had filled data submission for all 2018 isolates. Hospital A is a broad general public University Hospital that exclusively attends the public health system (Sistema Único de Saúde–SUS) with 500 beds. It also has many ICU beds (83) and different transplantation wards, including Bone Marrow Transplant (BMT). Hospital B is a private hospital with 116 beds that receive patients for private services and also from health insurance companies. It is a Hospital dedicated to Class A patients that operates mainly elective orthopedics surgeries and has 30 ICU beds. Hospital C attends private and health insurance services and has 242 beds. Although it has a large obstetrics ward, it also has three ICU (NICU, Cardiac and General), summing up to 50 beds, a BMT and Hematology ward, and a variety of other clinical departments.

**Figure 6:**
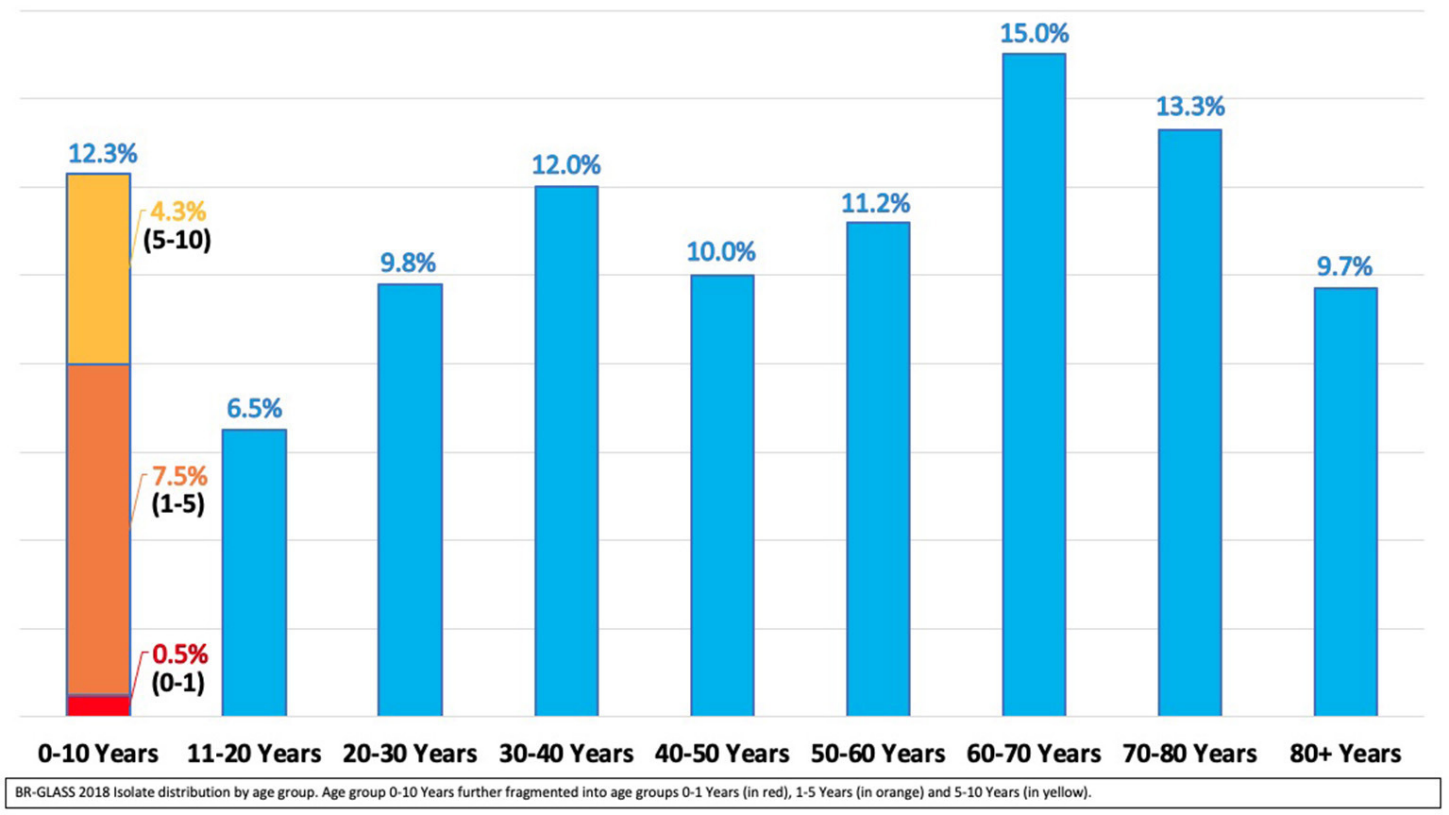
BR-GLASS 2018 Isolate distribution by age group.

Compiled data represents 11,347 isolates and 200,874 individual antimicrobial susceptibility test results for all drugs tested (media of 17–18 drugs tested by isolate). Regarding its origin 56.3% (6,397/11,347) were community-borne and 43.6% (4,950/11,347) hospital-borne. Females represented 58.0% of the origin of total collected samples, with the other 42.0% from males. Figure 6 shows the distribution of the isolates by age group.

Urinary tract samples were the most common topography, representing 56.1% of isolates (6,372/11,347), followed by blood samples, 18.3% of isolates (2,072/11,347), and respiratory tract samples, 10.9% of isolates (1,238/11,347).

In both populations (community and hospital), bacterial isolates showed predominance of Gram-negative bacteria constituting 65.2% (7,394/11,347) of total isolates. The most prevalent species was *E. coli* representing 32.1% of isolates (3,643/11,347), followed by Coagulase Negative *Staphylococci*—CoNS (12.5%; 1378/11,347), *S. aureus* (12.1%; 1369/11,347), *K. pneumoniae* (8.5%; 965/11,347) and *Enterococcus faecalis* (7.5%; 855/11,347). When considering only isolates originated from hospitals the most prevalent bacteria were CoNS (22.4%; 1,110/4,950), *E. coli* (18.2%; 901/4,950), *S. aureus* (15.2%; 752/4,950), *K. pneumoniae* (12.5%; 621/4,950) and *E. faecalis* (9.9%; 491/4950).

When observing the body systems in both groups (outpatient and inpatient), *E. coli* was the most prevalent bacteria in urinary tract samples (3591/ 6372; 56.4%). Coagulase-negative *Staphylococci* prevailed in blood samples (856/2072; 41.3%) and *S. aureus* in respiratory tract samples (637/1238; 51.4%). However, when filtering results for inpatients only, the most frequent isolates for each material are *E. coli* (572/1,211; 47.2%) for urinary tract samples, Coagulase-negative *Staphylococci* (371/1,746; 21.2%) for blood samples and *P. aeruginosa* (185/526; 35.2%) for respiratory tract samples.

When analyzing compiled drug susceptibility results from all strains, 141,648 (70.5%) results from AST were susceptible; 9,950 drugs (4.9%) gave an intermediate result, and 49,276 (24.5%) antimicrobials gave a resistant result on AST. Resistant results upscale to 30.8 % when observing inpatients only and it drops to 19.0% when watching outpatients only. The microorganism showing the worse susceptibility profile was *A. baumannii*, with 34.7% of susceptibility to all drugs tested, followed by ESBL-producing *K. pneumoniae* with 41.0% overall susceptibility results.

The three most commonly tested antimicrobials were ciprofloxacin, the aminoglycoside gentamicin, and sulfamethoxazole-trimethoprim. Their resistance rates were 29.8, 16.7, and 32.1%, respectively. The most effective drugs tested were vancomycin (1.2% resistant) and linezolid (2.8% resistant) for gram-positive bacteria. While gram negatives were more susceptible to polymyxin/colistin (6.9/2.5% resistant) and fosfomycin (8.1% resistant). Other important drugs were: amikacin, with a 9.7% overall resistant rate that rises to 11.5% for inpatient population; meropenem (17.1–21.7% resistance in all patient *vs*. inpatient only); ceftriaxone (19.2–34.8%–resistance in all patient *vs*. inpatient only); cefepime (27.7–38.1% resistance in all patient *vs*. inpatient only). Figures 1–5 shows more details about these results.

## Discussion

The BR-GLASS Program is considered to be an intelligence apparatus in the Brazilian health system to fight AMR. Therefore, this study consists of the first report of the results. Also, these findings were compared to similar data from comparable foreign environments and with data from similar national studies. These allow BR-GLASS data to be plotted in the world AMR map, conferring a single opportunity, as we discuss here, to control and prevent the expansion and development of antimicrobial resistance.

Throughout this study, we can see a clear predominance of Gram-negative bacteria, with more than 65% of isolates from both inpatients and outpatients. Regarding community-acquired isolates, we had no surprise, with most of the samples being urine-born *E. coli*. Nevertheless, it is interesting to state that CoNS and *S. aureus* had a critical percentage, reaching almost 25% of the total isolates.

For the most common community-acquired bacteria (*E. coli*), it is essential to point the ciprofloxacin resistance that grows steadily, reaching up to 32%.

**Figure 7:**
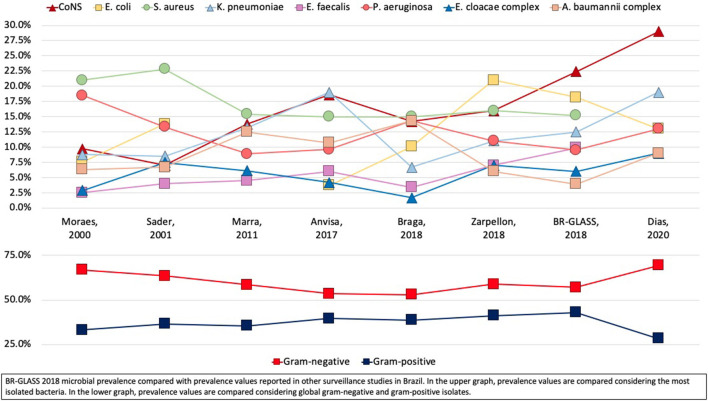
BR-GLASS 2018 microbial prevalence compared with prevalence values reported in other surveillance studies in Brazil.

Although 56% of the collected data refers to community-borne bacteria, we will focus our analysis on hospital-associated infections. Within this group, Gram-negative still prevails, with 57% of the total isolates. When we determine the frequency of the main hospital-associated species isolated in BR-GLASS and compare it to extensive Brazilian studies in the last 20 years (see Figure 7) (3, 10–14), we can observe the consolidation of the ESKAPE group, as stated by Rice (5). As years go by, this group was joined by two others, initially not so resistant: *E. coli* and Coagulase-negative *Staphylococci* (CoNS). This fact brings in a new perspective to the ESKAPE group, with *E. coli* and CNS becoming increasingly resistant, as we discuss later.

In a sizeable Brazilian study analyzing 23,935 hospital isolates during a 6 years-period (2012–17), CoNS was the most isolated, similar to our findings (Dias, 2020—submitted). Alternatively, *Klebsiella pneumoniae* (19.0%) was the second most common isolate with *E. coli* (13%) and *P. aeruginosa* (13.5%) in third place, while in our study *E. coli* took second place, followed by *S. aureus* and *K. pneumoniae*–see Figure 7. Although the study by Dias et al. (2020, submitted) does not point the exact prevalence of gram positives by separate species, we can assume that the stated 29% of GP isolates are represented mainly by CoNS, *S. aureus*, and *Enterococcus* spp, as it is also seen in most studies in Brazil. In contrast with those findings, our research shows a prevalence of 49.4% for the main gram-positive species. Such higher prevalence in our study may be explained by the method of data compilation. BR-GLASS does not make clinical correlations to address the exact etiology of the infection. So, in our case, many CoNS could be attributed to contamination by skin microflora during the sample collection procedure.

Zarpellon et al. established a Surveillance Program in a teaching hospital during the period from 2012 to 2014 in Southern Brazil (Maringa, PR) (10). The most common isolates from inpatients were almost the same as our study, with *E. coli* corresponding to 21%, *S. aureus* 14%, and *CoNS* 13%, with only one difference–the percentages of CoNS, that represented 22% of inpatient isolates in our study. This finding might reflect the variation from hospitals and the patient's status. Our understanding is that our data is more recent (2018) than Zarpellon's (2012–2014), and invasive procedures are being increasingly used (especially catheter use), which favors CoNS infection.

Other studies in Brazil show similar findings. According to ANVISA–The National Health Regulatory Agency from Brazil, CoNS was the most frequent isolate from ICUs in 2017, representing 18.6 % of the total isolates (15).

Braga et al. (11) found a very similar percentage within the three most common isolates (*S. aureus, P. aeruginosa*, and CoNS), ranging from 14.2 to 15.0%. With *P. aeruginosa* presenting a much higher percentage than in our study.

### The ESKAPE Group— *Escherichia coli*

*Escherichia coli* is a known gram-negative uropathogen in community-acquired urinary tract infections. Also, it was responsible for a significant part of healthcare-associated urinary tract infections, representing 47.2% of inpatient UTIs. These results corroborate data shown by Sader et al. (47.6%) (13) and contrast with data revealed by De Carvalho and Gontijo Filho −18.2% (16) and Braga et al. 28.6% (11). *Escherichia coli* has a considerable presence in the hospital microbiome and, therefore, may be an important vector in AMR transfer, especially considering their effectiveness in both receiving and distributing genetic determinants of resistance (17). *Escherichia coli* commonly exhibit genes responsible for β-lactam resistance such as ESBL (18). In Brazil, it often carries New Delhi Metallo-beta-lactamase (NDM) gene (19) or even the mobilized colistin resistance (*mcr*-1) gene (20).

**Figure 8:**
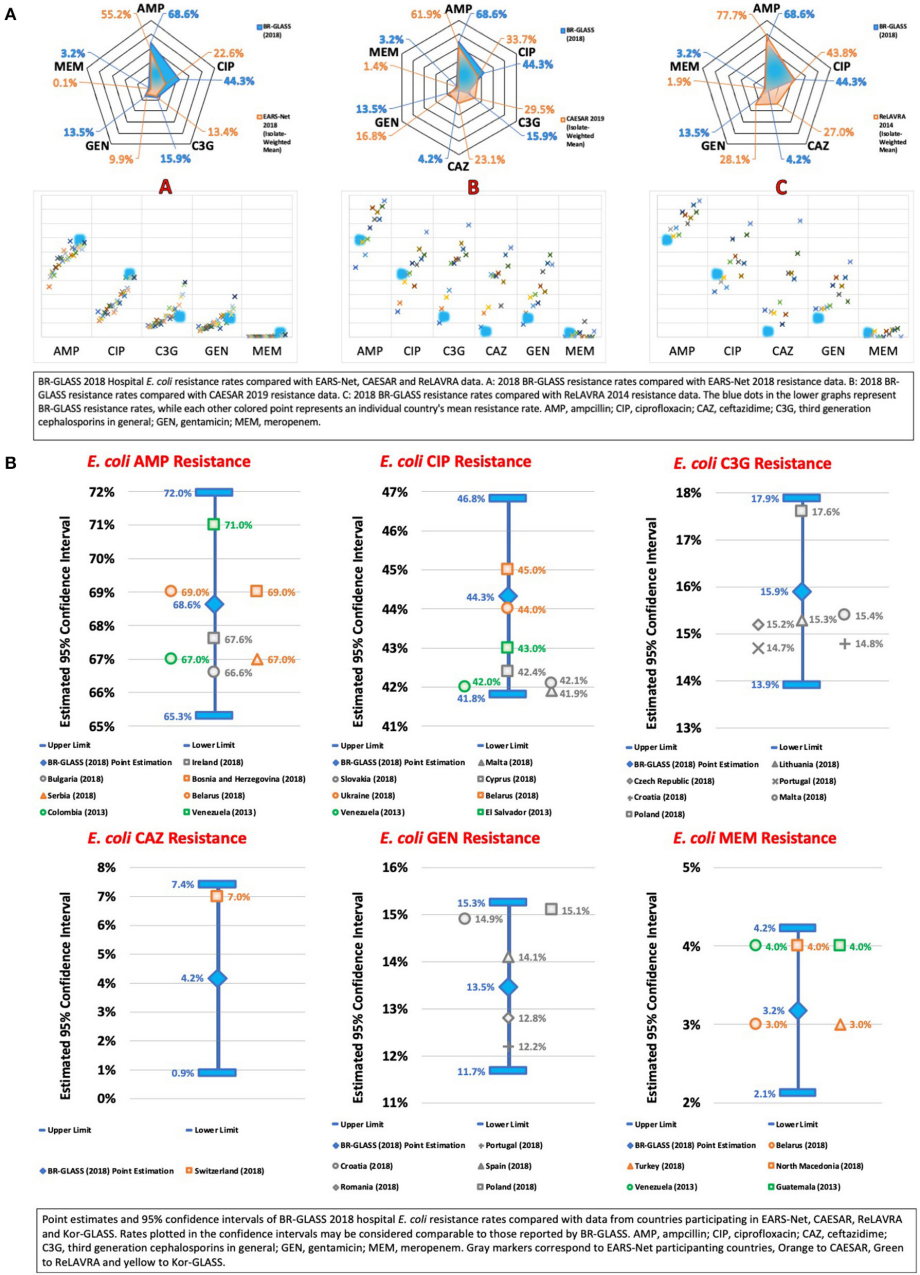
**(A)** BR-GLASS 2018 Hospital *E. coli* resistance rates compared with general EARS-Net, CAESAR and ReLAVRA data. **(B)** BR-GLASS 2018 Hospital *E. coli* estimated resistance rates with 95% confidence intervals and comparable rates reported in different countries.

We verified high *E. coli* resistance to ampicillin (68.6%). It may be suggestive of high plasmidial *AmpC* prevalence amongst the Brazilian nosocomial *E. coli* population. Such a rate was higher than that reported by Sader et al. (57.8%) (13). Probably because of an increase in the transfer of such resistance genes between these bacteria. In comparison with international results, our data showed a higher than the calculated average (60.6%) resistance to ampicillin (see Figure 8).

We observed in our study that inpatient *E. coli* showed a remarkable resistance rate to ciprofloxacin (44.3%), potentially leading to treatment failure (21). Such a rate was consistently higher than reported by Sader et al. (10.9%) (13) and by De Carvalho and Gontijo Filho (31.2%) (16). Massive fluoroquinolone empirical prescription could be the reason for this increase (22). In comparison with international results, our data showed a higher than the calculated average (28%) resistance to ciprofloxacin (see Figure 8).

We verified low resistance to ceftriaxone (15.9%) and ceftazidime (4.2%) for *E. coli*, with rates comparable (14.7%) and lower (26.1%) than the calculated average, respectively. In comparison with other Brazilian studies, our rate (15.9%) was double that reported by Sader et al. (7.4%) (13), but lower than rates reported by De Carvalho and Gontijo Filho (18.7%) (16). These resistance levels indicate a low prevalence of extended-spectrum β-lactamase (ESBL) gene. The most common of which is CTX-M-2 in *E. coli* in Brazil. Generally, it results in treatment with carbapenems (13), which may lead to increasing selective pressure toward carbapenemase-producing *E. coli*. As a consequence, ESBL could be a factor in increasing carbapenem-resistance strains.

*Escherichia coli* resistance to gentamicin was also low (13.5%) but still higher than the data presented by Sader et al. (7.4%) (13). Our rates were comparable to the calculated average (14.3%).

As previously stated, carbapenem resistance in *E. coli* is worrisome due to the combination of the importance of carbapenems in treating multidrug-resistant gram-negative bacteria infections (13). Also, due to the versatility of *E. coli* in transferring resistance genes (23). Consequently, it may be implied that carbapenem resistance is an interesting metric for both tracking general AMR and evaluating the risks it may bring to health systems. Our 3.2% resistance rate toward meropenem is, therefore, unsettling, especially considering that Sader et al. reported a rate of 0% in Sader et al. (13). Internationally, such rate is considerably higher than average (0.5%) For more data, see Figure 8.

### The ESKAPE Group—*Klebsiella pneumoniae*

*Klebsiella pneumoniae* is a widely distributed and relatively lethal gram-negative pathogen. *Klebsiella pneumoniae* BSIs, in general complications of primary LRTI or UTI, present mortality rates ranging from 27.4 to 37% (24). The rapid spread of AMR amongst these bacteria and subsequent treatment failure is, therefore, a significant cause of concern which motivates the necessity for the surveillance of their resistance patterns. Carbapenem-resistance is of particular interest due to the first line use of this class of antimicrobial agents in treating ESBL-producing *K. pneumoniae* infections (25). As a consequence, physicians need to use tigecycline and polymyxins (23). Consequently, the possibility of an increase in Carbapenem-resistant *Enterobacteriaceae* (CRE), specially Carbapenem-resistant *K. pneumoniae* (CRKP), is both likely and worrisome.

Although carbapenemase-producing *K. pneumoniae* is the primary cause of concern, it does not diminish the significant importance of ESBL-producing *K. pneumoniae* in the clinical setting. Therefore, it may be of interest to maintain adequate surveillance and compare cephalosporin resistance with carbapenem resistance, to appropriately measure the prevalence of ESBL-producing non-carbapenem-resistant *K. pneumoniae* (26).

**Figure 9:**
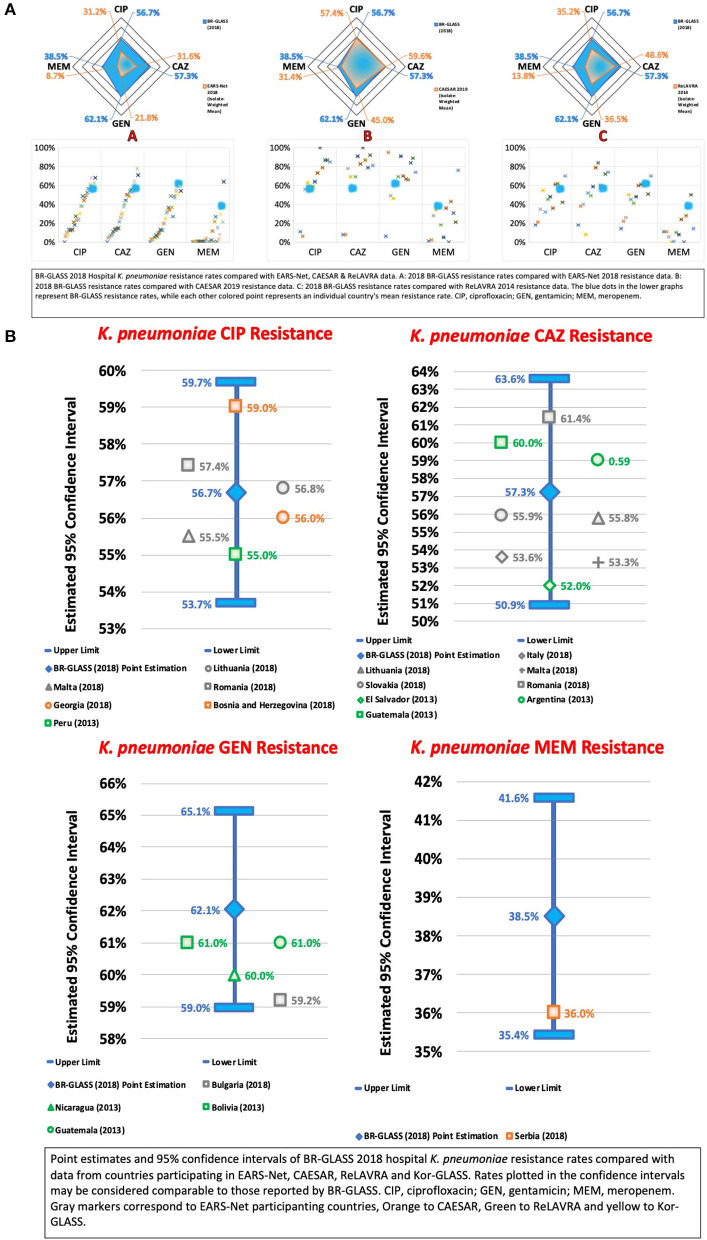
**(A)** BR-GLASS 2018 Hospital *K. pneumoniae* resistance rates compared with general EARS-Net, CAESAR, and ReLAVRA data. **(B)** BR-GLASS 2018 Hospital *K. pneumoniae* estimated resistance rates with 95% confidence intervals and comparable rates reported in different countries.

Therefore, our study found a high resistance rate of ceftazidime resistance (57.3%) which, when compared with national studies, is higher than reported by Sader et al. (30.9%) (13) and similar to rates shown by De Carvalho and Gontijo Filho (57.6%) (16) and by Marra et al. (54.4%) (12). Compared with international results, our rates were higher than the calculated average (39.7%) (see Figure 9).

Accordingly, we verified an impressive growth of *K. pneumoniae* meropenem resistance from 0.03% reported by Sader et al. (13) 19 years ago and 1.3% reported by Marra et al. (12) 9 years ago, up to 38.5% in our results. Both are to be expected since the prevalence of carbapenemase-producing *K. pneumoniae* began its growth in the 2010s. In Brazil, the first CRKP phenotypes were isolated in São Paulo in 2003 and 2005, with the first being IMP-1-producing and the second being *K. pneumoniae* carbapenemase 2 (KPC-2)-producing in Recife, in 2006 (23, 27). While the IMP-1 phenotype remained restricted to São Paulo, the KPC-2 phenotype rapidly disseminated in the 2010s and became endemic in Brazil, with carbapenem-resistance increasing from 6.8% in 2011 to 35.5% in 2015, with 96.2% of the resistant phenotypes being KPC-2-producing (28). Simultaneously, carbapenem-resistant KPC-2-producing *K. pneumoniae* dominated the CRE composition in Brazil, coming from just 17% in 2010 to an impressive 82% of CRE associated HAIs in 2011 (29). This expansion of the KPC-producing *K*. *pneumoniae* does not show signs of slowing down, with the rate still growing and continuously amplifying their presence in Brazil and Paraná. Here, out of 942 CRE sent to a reference lab, *K. pneumoniae* represented 94.3% of the isolates that expressed bla_KPC−2_ (30, 31).

Still, our carbapenem-resistant results were also higher than the rates reported by Zarpellon et al. (25.1%) (10) and comparable to the rates reported by Bartolleti et al. (35.5%) (28). Considering that Bartolleti et al. collected data from the epicenter of the CRKP epidemic in Brazil, it may be suggested that our country is experiencing a progressive expansion in the prevalence of such pathogens that radiated outwards from São Paulo, only recently reaching comparable resistance rates.

Internationally, our carbapenem resistance rates were consistently higher than the calculated average (12.6%). Such a high rate corroborates the progressive and significant expansion of KPC-2-producing *K. pneumoniae* but also suggests when compared with the ceftazidime resistance rates, the presence of a considerable amount of carbapenemase–negative ESBL-*K. pneumoniae* in the Brazilian AMR scenario (see Figure 9).

Considering the possible persistence of the carbapenem-susceptible ESBL-producing *K. pneumoniae*, we also analyzed resistance rates to ciprofloxacin, a drug effective against such bacteria (13). We found a resistance rate of 56.7%, much higher than 4.5% reported by Sader et al. (13), higher than 36.2% reported by Marra et al. (12) and comparable to 56.8% reported by Zarpellon et al. (10) 2 years ago. Such a historical pattern of growing ciprofloxacin resistance may be a result of selective pressure produced by ciprofloxacin use in place of carbapenems against ESBL-producing *K. pneumoniae*, due to the threat of KPC-2-producing *K. pneumoniae*, and the previous use of this drug (18).

Our results of ciprofloxacin resistance rates were shown to be higher than the calculated average (35%).

Also, we verified *K. pneumoniae* resistance to gentamicin, to which KPC-producing *K. pneumoniae* may occasionally be susceptible to, allowing for monotherapy using this drug (32). We observed a resistance rate of 62.1%, higher than rates reported by Sader et al. (39.3%) (13), by Marra et al. (30.7%) (12), and by Zarpellon et al. (55.5%) (10). Our results show higher than the calculated average (28.9%) resistance. This would imply that higher carbapenem resistance rates would correlate with higher gentamicin resistance rates, as is observed in this study. As in the case of ciprofloxacin, an alternative drug for ESBL and KPC-*K. pneumoniae*, gentamicin also presents a similar historical loss of effectiveness, possibly due to increases in the use of gentamicin promoting selective pressure toward its resistance. For more data, see Figure 9.

### The ESKAPE Group—*Acinetobacter baumannii*

The genus *Acinetobacter*, in particular, its most successful member *A. baumannii*, represents one of the significant threats to the antibiotic era, with pan-resistant phenotypes having already been reported (33). A gram-negative pathogen with a diverse and rapidly developing resistance mechanisms allied with notable hospital environment survivability, *A. baumannii* is not only highly adapted to modern healthcare infrastructure but also highly prevalent, causing ~20% of ICU infections worldwide (34). Of particular concern are multidrug-resistant *A. baumannii* (MDR-Ab) and carbapenem-resistant *A. baumannii* (CRAB), with the latter posing an extraordinarily complex situation considering carbapenems were, until recently, the drug of choice in treating infections caused by multidrug-resistant *Acinetobacter spp*. (35).

There is a large body of evidence that supports that *A. baumannii* is critical in Brazil's general AMR scenario, with origins in the early 2000s. The first outbreak by MDR *A. baumannii* OXA-23 was reported by Dalla-Costa in Parana's capital Curitiba in 2003, referring to an episode from two hospitals in 1999 (36). Toledo et al. (29) described that Carbapenem-Resistant *A. baumannii* (CRAB) was responsible for 34.5 to 36.2% of hospital-acquired pneumonia during the years of 2010 to 2011. *bla*_OXA−23−like_ is highly expressed amongst the Brazilian CRAB population, being present in up to 100% of isolates (37), as well as in all Latin America (35). Along with OXA-23-like, NDM has also been detected in Brazilian *Acinetobacter* spp (38, 39). Consequently, the sum of these multiple resistance-conferring factors creates a scenario in which *Acinetobacter* resistance is poised to maintain its progressive expansion.

**Figure 10:**
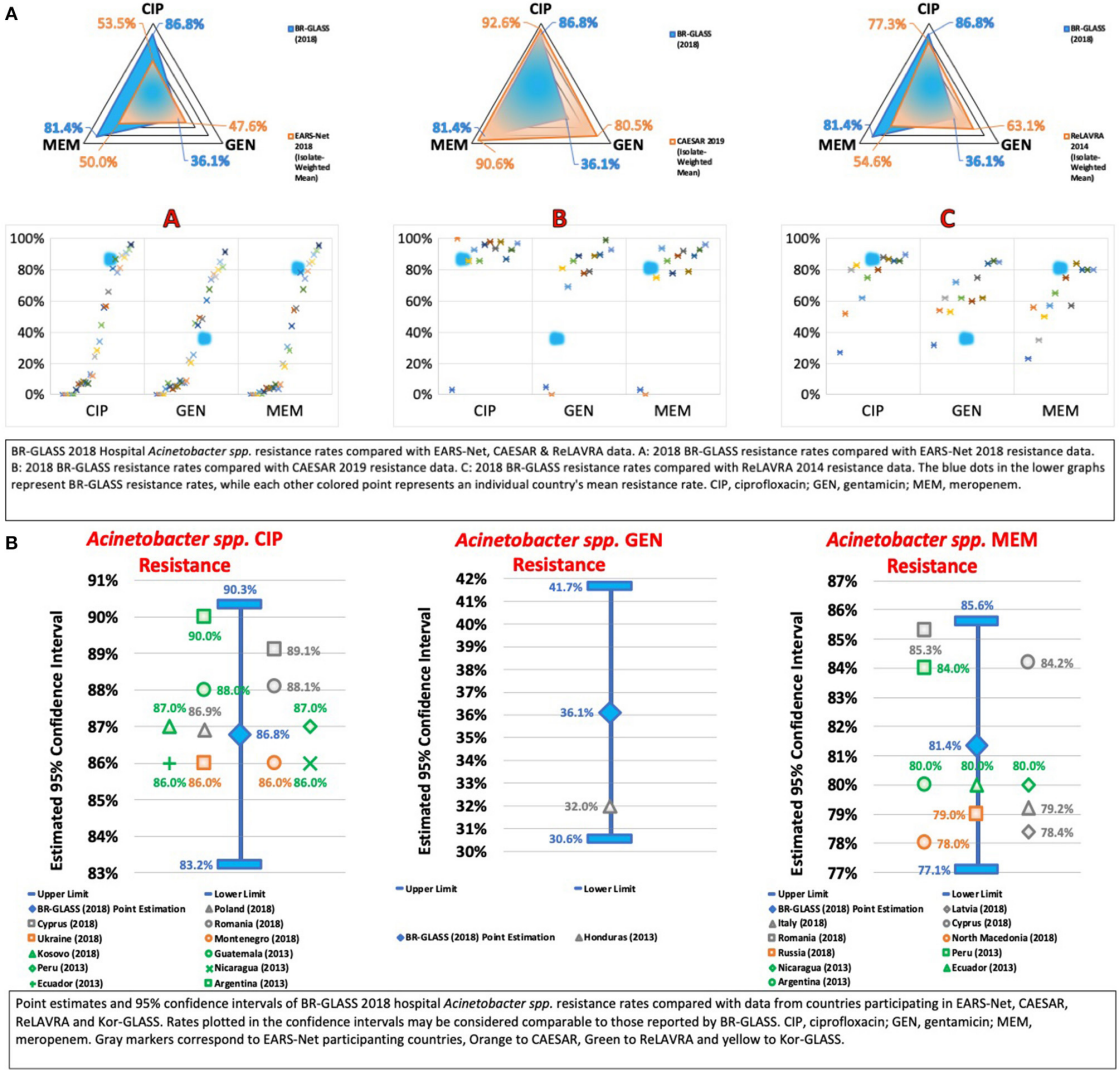
**(A)** BR-GLASS 2018 Hospital *Acinetobacter spp*. resistance rates compared with general EARS-Net, CAESAR, and ReLAVRA data. **(B)** BR-GLASS 2018 Hospital *Acinetobacter spp*. estimated resistance rates with 95% confidence intervals and comparable rates reported in different countries.

Accordingly, we evaluated the *Acinetobacter spp*. resistance to meropenem to determine the current situation in terms of CRAB. We found a high resistance rate of 81.4%, expressively higher than 8% reported by Sader et al. (13), 25% reported by De Carvalho and Gontijo Filho (16), 56.4% reported by Marra et al. (12) and comparable to 77.6% reported recently by Zarpellon et al. (10). Therefore, we notice a historical pattern of progressive growth in resistance rates that accompanies the timeline of the detection of genetic determinants of resistance. Compared to international data, our resistance rate is higher than the calculated average (60.5%) (see Figure 10).

To evaluate MDR-Ab in general, we analyzed resistance to ciprofloxacin. We observed a high resistance rate of 86.8%, higher than the 55.5% reported by Sader et al. (13) and 73.4% reported by Marra et al. (12). However, similar to the 86.2% reported by Zarpellon et al. (10). This previous study's data confirms that general MDRr-Ab preceded CRAB in Brazil. Nevertheless, it also corroborates the hypothesis that the advent of carbapenemase production in *Acinetobacter spp*. expanded antibiotic resistance in general within the genus. Internationally, our ciprofloxacin resistance rate is higher than the calculated average (72.7%) - see Figure 10.

In terms of gentamicin, we found a low resistance rate of 36.1%, lower than 48.2% reported by Sader et al. (13) 19 years ago, 51.8% reported by Marra et al. (12), 9 years ago and 79.6% reported by Zarpellon et al. (10). This was surprising, especially considering the apparent trend of increasing gentamicin resistance being established by previous studies and deserves further investigation to clarify the importance gentamicin has in MDR-Ab and, possibly, CRAB. Motivated by this, we analyzed amikacin resistance rates. We found a rate of 63%, higher than 57.7% reported by Sader et al. (13) and lower than 77% reported recently by Zarpellon et al. (10). This result is also puzzling and raises the question: could aminoglycoside susceptibility in MDR-Ab and CRAB, in general, be increasing? Compared with international results, our gentamicin resistance rate was also notably lower than the calculated average (61.4%). For more data, see Figure 10.

### The ESKAPE Group— *Pseudomonas aeruginosa*

*Pseudomonas aeruginosa* is a critical gram-negative opportunistic pathogen in the nosocomial setting, especially in LRTIs in immunocompromised patients (40, 41). Our results corroborate such a high prevalence of *P. aeruginosa* in LRTIs, with these bacteria being the primary etiologic agent in hospital LRTIs, representing 35.2% of cases, a lower proportion than that reported by De Carvalho and Gontijo Filho (42%) (16). In addition to its prevalence, *P. aeruginosa* is also a highly adaptable pathogen, capable of continuously surviving in the hospital environment and possess mechanisms of intrinsic and versatile resistance, such as inducible AmpC expression (41). Considering its high prevalence and environmental fitness, the expansion of AMR in these bacteria is worrisome.

Among multidrug-resistant *P. aeruginosa*, carbapenem-resistant *P. aeruginosa* (CRPA) is of significant note due to few therapeutic options left, like polymyxins (41) Among MDR isolates, CRPA has been shown to be responsible for 14.9% of LRTIs (29), which shows that although not as prominent as CRAB or *K. pneumoniae*, CRPA is a potentially troublesome bacteria in the current AMR scenario.

**Figure 11:**
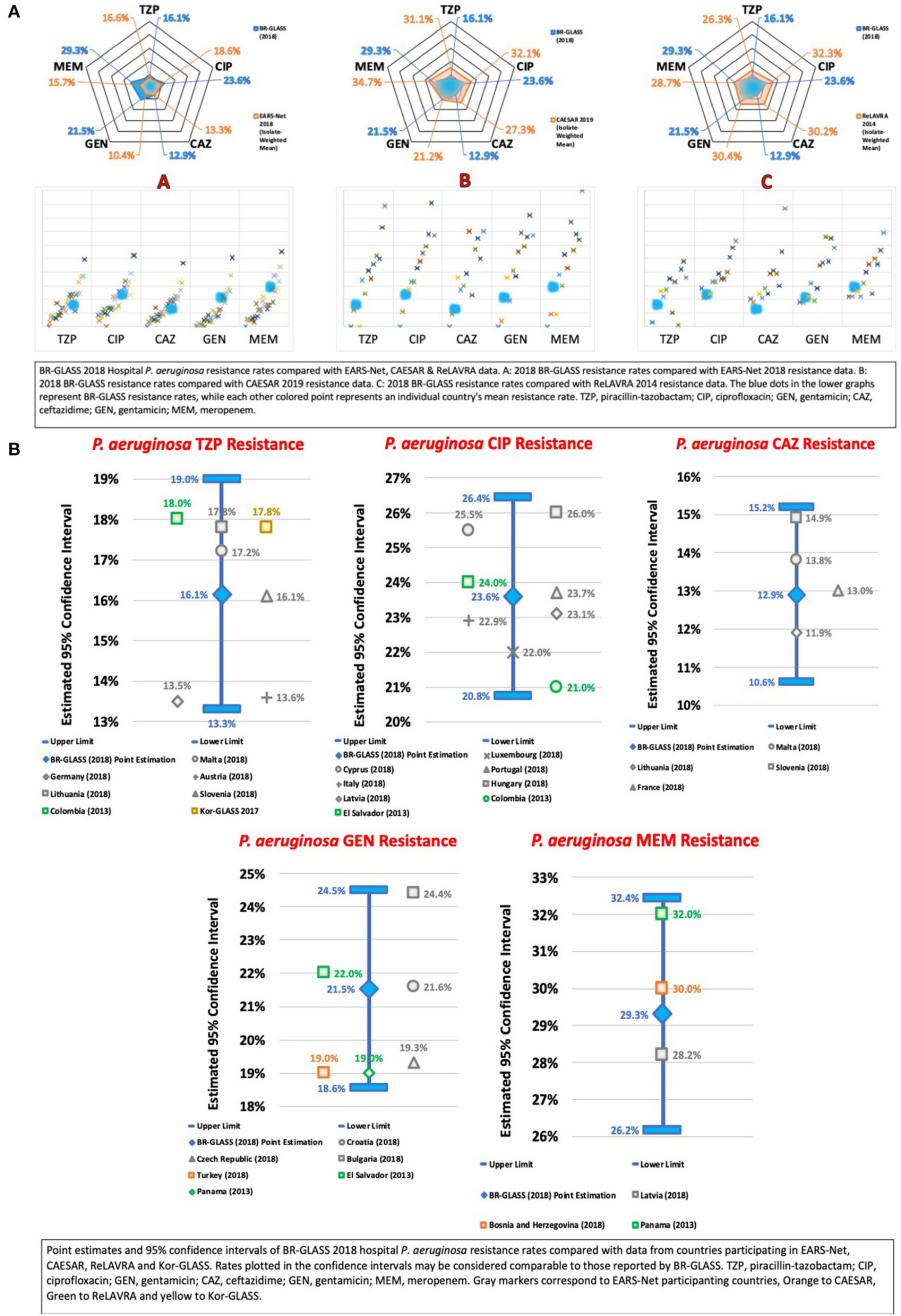
**(A)** BR-GLASS 2018 Hospital *P. aeruginosa* resistance rates compared with general EARS-Net, CAESAR, and ReLAVRA data. **(B)** BR-GLASS 2018 Hospital *P. aeruginosa* estimated resistance rates with 95% confidence intervals and comparable rates reported in different countries.

Accordingly, we assessed meropenem resistance in *P. aeruginosa*. We found a rate of 29.3%, higher than the rate reported by Sader et al. (17.3%) (13) and lower than the rates reported by De Carvalho and Gontijo Filho (71.7%) (16), by Marra et al. (35.8%) (12), and by Zarpellon et al. (38.9%) (10). In comparison with international results, our rates were higher than the calculated average (22.4%) - see Figure 11.

We analyzed *P. aeruginosa* resistance to the 3GC ceftazidime. We observed a resistance rate of 12.9%, lower than rates reported by Sader et al. (33.3%) (13), by De Carvalho and Gontijo Filho (71.7%) (16), and by Marra et al. (36.6%) (12). Internationally, our results were lower than the average rate (21.7%). Comparing ceftazidime to meropenem susceptibility (12.9 ×29.3%), one might think these findings are awkward. However, according to Pournaras et al., these results are very common in regions where overexpression of the efflux pump is frequent (42). Another study by Kalluf et al. support this evidence, since only 25% (41/161) CRPA were positive for *bla*_SPM_ (43). At our lab, we have only a 25% positivity rate for MBLs in CRPAs (Marcelo Pillonetto—unpublished results), which leads to the hypothesis that in our state carbapenem resistance in *P. aeruginosa* is caused mainly by down-regulation or porin loss and/or efflux pumps, as also stated by Campana et al. (44) and Xavier et al. (45).

We found a piperacillin-tazobactam resistance rate of 16.1%, lower than the rate reported by Sader et al. (26%) (13) and by Marra et al. (33.9%) (12). Our rate was lower than the calculated average (21.8%) - see Figure 11.

The ciprofloxacin resistance rate in *P. aeruginosa* was 23.6%. Lower than the rate shown by Sader et al. (32.7%) (13), by De Carvalho and Gontijo Filho (76.1%) (16), by Marra et al. (45.6%) (12) and by Zarpellon et al. (40.1%) (10). Compared with international results, our rate was comparable to the calculated average (25.5%). These findings are interesting, especially if we consider the consistently higher ciprofloxacin-rates found by this study in *K. pneumoniae* and *A. baumannii* (56.7 and 86.8%, respectively).

We also analyzed gentamicin resistance and detected a resistance rate of 21.5%, lower than rates observed by Sader et al. (38%) (13), by Marra et al. (45.7%) (12) and by Zarpellon et al. (38.9%) (10). Internationally, our results were comparable to the calculated average (19.9%).

The overall resistance of *P. aeruginosa* in our study is surprisingly less than expected compared to publications cited above, leaving some questions to solve that are beyond the scope of this report. For more data, see Figure 11.

### The ESKAPE Group— *Staphylococcus aureus*

*Staphylococcus aureus* is a critical gram-positive pathogen in human infection, with our data showing such significance in its predominance in general (outpatient and inpatient) lower respiratory tract infections (LRTI), being the etiologic agent of 51.4% of these cases. De Carvalho and Gontijo Filho analyzed the prevalence of such bacteria in HAIs. It was responsible for 30% of LRTIs and 19.3% of bloodstream infections (BSI), representing the second most isolated pathogen in both types of nosocomial infections (16). Resistance in *S. aureus* thus is a matter of concern, due to its ubiquitous presence in both the community and hospital settings.

Considering that the treatment of *S. aureus* infections is dependent on antibiotics, the continuous monitoring of fast-developing resistance to amply used antimicrobials is fundamental in preventing treatment failure (46). In the matter of surveillance in clinical practice, *S. aureus* can be separated into two clinical phenotypes according to effective treatment options available: methicillin-susceptible *S. aureus* (MSSA) and methicillin-resistant *S. aureus* (MRSA). MRSA can be sub-divided in two other groups: non-multiresistant MRSA (nmrMRSA) —which is resistant to less than three antimicrobial groups and multiresistant MRSA (mrMRSA), which is resistant to three or more antimicrobial groups (47). High MRSA rates are worrisome. Especially when considering that multiresistant MRSA represents 20.4% of BSIs, 27.5% of surgical site infections (SSIs), and 16.6% of LRTIs (29). *Staphylococcus aureus* infection poses a risk that should be closely monitored (48).

**Figure 12:**
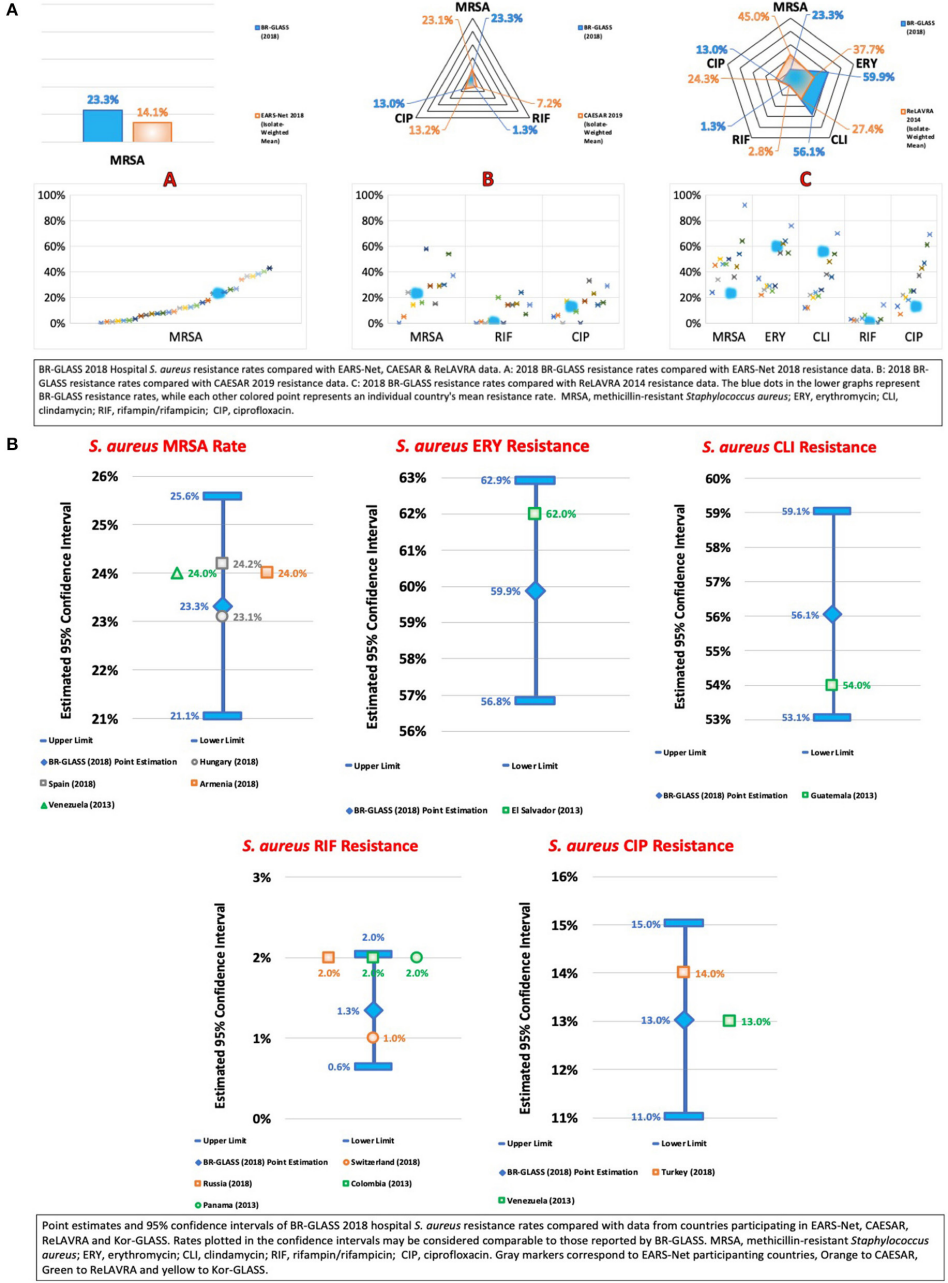
**(A)** BR-GLASS 2018 Hospital *S. aureus* resistance rates compared with general EARS-Net, CAESAR, and ReLAVRA data. **(B)** BR-GLASS 2018 Hospital *S. aureus* estimated resistance rates with 95% confidence intervals and comparable rates reported in different countries.

Accordingly, our data showed that ~23.3% of Brazilian *S. aureus* were MRSA, a rate lower than that shown by Sader et al. (34%) (13), by De Carvalho and Gontijo Filho (60%) (16), by Marra et al. (43.7%) (12), and by Zarpellon et al. (> 44%) (10). These results may suggest that Brazilian *S. aureus* rates may be lower than previously reported. In comparison with international findings, our resistance rate is slightly higher than the calculated average (19.9%) - see Figure 12.

Before discussing resistance among treatment options for MRSA, we also chose to analyze erythromycin resistance among *S. aureus*, a macrolide antibiotic with activity against 85% of MSSA, and 65–75% of non-multiresistant MRSA (47). Therefore, it is a possible treatment option for less resistant phenotypes of these bacteria. We found a high erythromycin-resistant *S. aureus* (ERSA) rate of 59.9%, slightly higher than the rate reported by Sader et al. (54.7%) (13). These high rates of ERSA associated with low rates of MRSA may be suggestive of higher resistance rates amongst Brazilian MSSA.

Clindamycin is an active antimicrobial agent against nmrMRSA and MSSA, mainly being a first-line treatment for soft tissue and skin infections caused by nmrMRSA (47). We also observed an elevated clindamycin resistance rate of 56.1%, higher than that reported by Sader et al. (33.5%) (13) and by Marra et al. (47.4%) (12). These findings corroborate the previous suspicion that Brazilian MSSA might be developing higher resistance without transitioning, necessarily, into the MRSA phenotype.

Rifampicin generally is used as a first-line treatment in combination with another drug such as fusidic acid or linezolid to treat mrMRSA infections (47). Our results showed a low rifampicin resistance rate of 1.3%, considerably lower than rates reported by Sader et al. (28.9%) (13) 19 years ago. This low result, along with previously discussed results, may be an indication of its low use in hospital settings in Brazil.

Ciprofloxacin is generally an antibiotic reserved for gram-negative bacterial infections. Still, rising resistance to first line mrMRSA therapies has brought it into the fold of mrMRSA treatments, commonly used in combination with other drugs in the manner of rifampin due to, similarly, the rapid development of resistance (47). Our results showed a ciprofloxacin resistance rate of 13%, lower than both rates reported by Sader et al. (34.4%) (13) and Marra et al. (38.2%) (12). Considering previously discussed data, this is coherent with our speculation that, in Brazil, the MRSA situation is not so complicated. Therefore, the use and consequent selective pressure toward ciprofloxacin is not an issue. For more data, see Figure 12.

### The ESKAPE Group—*Enterococcus faecium*

*Enterococcus faecium* is an intrinsically resistant pathogen associated with bacterial endocarditis in the nosocomial setting. It rapidly spreads by cross-contamination to patients in outbreaks (49). To treat bacterial endocarditis caused by *Enterococci*, antimicrobial therapy must be successful. Considering the extensive intrinsic resistance *E. faecium* presents, drug therapy is already challenging in standard settings, becoming even more so in cases of increased resistance, such as in vancomycin-resistance. In scenarios of infection by vancomycin-resistant *Enterococcus* (VRE), of which *E. faecium* makes up a significant part of, the physician becomes dependent on either non-reliable treatment options or treatment options with toxic side effects (49). Consequently, surveillance of VRE and ampicillin-resistant *E. faecium* is relevant in preventing lethal treatment failure and monitoring outbreaks.

In Brazil, VRE infections were first reported in 1998 by Dalla-Costa et al. (50), although never becoming truly endemic, generally resurging in sporadic outbreaks (51). Our data appears to corroborate this and also supports the observations by Panesso et al. that VRE prevalence is low among clinical isolates. Possibly, even furthering their hypothesis that Latin American VRE expansion is delayed in comparison to the United States (52, 53).

In terms of resistance, we analyzed both ampicillin and vancomycin rates. Concerning ampicillin resistance, we observed a rate of 65.4%, higher than 5.6% reported by Sader et al. (13) and 21.7% reported by Marra et al. (12). It is important to cite that both studies analyzed general *Enterococcus spp*. resistance to ampicillin, which suggests higher *E. faecium* rates than those above.

**Figure 13:**
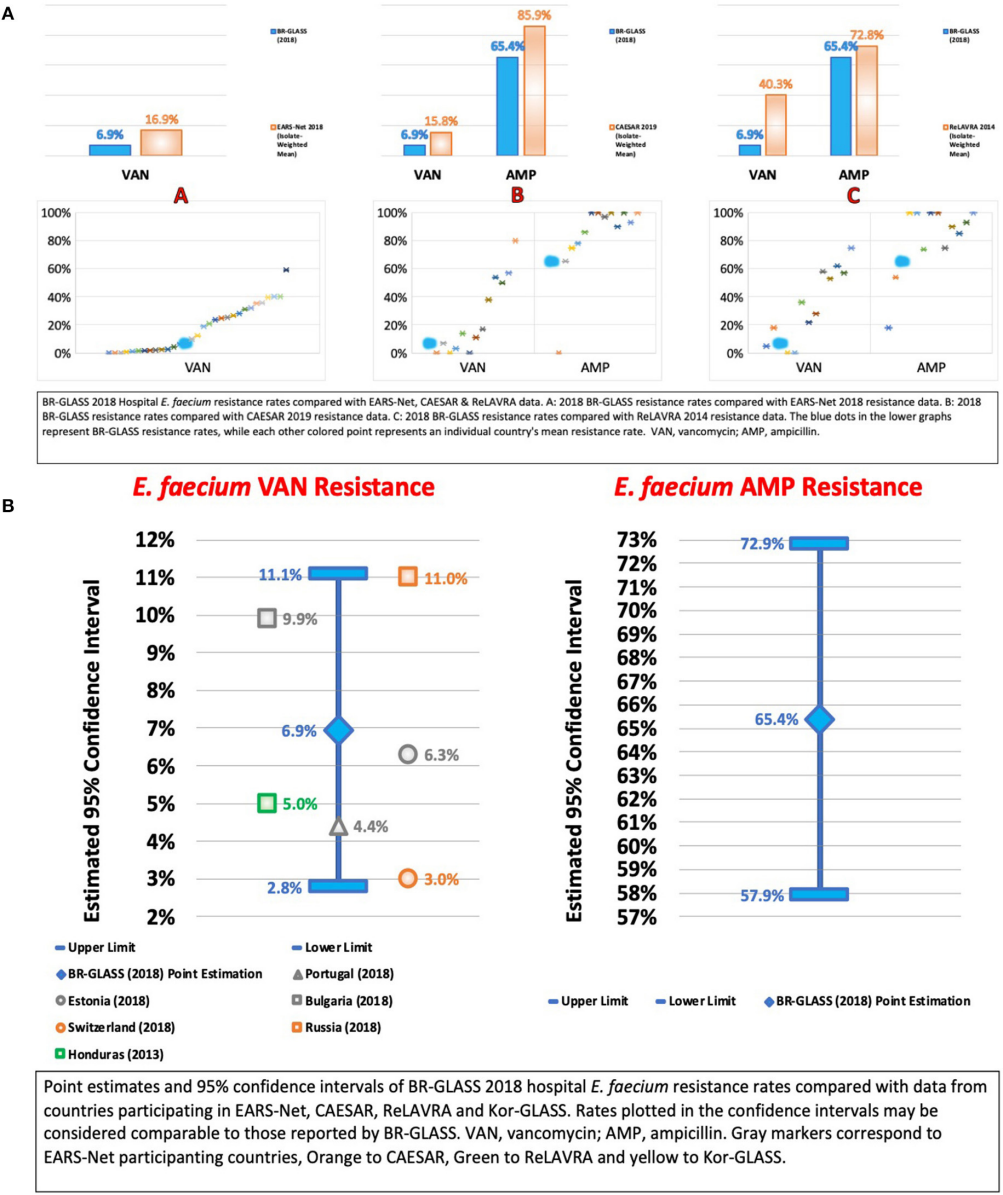
**(A)** BR-GLASS 2018 Hospital *E. faecium* resistance rates compared with general EARS-Net, CAESAR, and ReLAVRA data. **(B)** BR-GLASS 2018 Hospital *E. faecium* estimated resistance rates with 95% confidence intervals and comparable rates reported in different countries.

Regarding vancomycin resistance, we report a rate of 6.9%, lower than the 55.6% reported by Marra et al. (12), for *E. faecium*. Comparing with international results, our rate is lower than the calculated average (19.7%). It may be that it is not Latin America that is delayed in terms of VRE expansion, but Brazil, potentially posing an exciting opportunity for antimicrobial stewardship initiatives. For more data, see Figure 13.

## Study Limitations

Since our study presents the findings from three hospitals from one State in Brazil, it does not represent the full picture of the country's resistant profile. Broader data will be available in the upcoming years. The comparison of our findings with other Brazilian and international studies has the limitations of the year that data was collected. Since this is a retrospective, multi-institution study, not all the species have been tested against the same antimicrobials. Also, polymyxin/colistin resistance was not accessed because only few results were available using the recommended method (broth microdiluiton test). It would be essential to address some of the hypotheses shown here against the antimicrobial consumption rates. However, this data was not available at this moment. Finally, we decide not to compare the resistance rates of Coagulase-negative *Staphylococci* and *Enterobacter* spp. because of the scarce data available from other studies, compared to the other member of the ESKAPE group. Instead, we included the analysis of *E. coli*, once its resistance to antimicrobials has become steadily increasing. It was not possible to discuss community-acquired infections and its resistance profile.

## Conclusion

Here we present the successful implementation of a project to deliver a National Antimicrobial Resistance Surveillance System in Brazil. Starting with three sentinel hospitals, we gathered data from 11,347 isolates during the year of 2018. The project will continue to include other institutions from different geographic regions in Brazil.

The most evident problem regarding the prevalence and also resistance in this study was gram-negative bacteria. It was found in 65.2% of the overall samples, with *E. coli* being the most common species (32.1%). Also, the most worrisome resistant strains were HAI of *A. baumannii* and *K. pneumoniae*, especially when observing carbapenem-resistance (81.4 and 38.5%, respectively). Fluoroquinolone resistance rates were also a problem with frequency as high as 44.5% in HAI by *E. coli*.

One notable exception was that CoNS was the most prevalent bacteria in HAI, with 22.4% of the total isolates.

Considering the overall resistance in the main four GN species studied, carbapenems still have one of the lower resistance rates (except for *A. baumannii*), followed by aminoglycosides. Polymyxin/colistin resistance was not accessed because of methodology limitation aspects. For the gram, positive bacteria vancomycin is still a very effective drug, and resistance to macrolides is fairly high in *S. aureus* strains from this study. Also, VRE had a very low prevalence (6.9%).

The overall resistance rates of the main species studied showed that in Brazil, the results are more comparable to Turkey and Greece, better than Bolivia but more worrisome than Spain or Sweden.

The purpose of the Brazilian Minister of Health is to continue to strengthen the program, giving support to clinicians and epidemiologists to access a more precise picture of community and hospital-associated infections. Also, targeting the trends in antimicrobial resistance, supporting public health policies as well as a better empirical prescription and improvement of Stewardship Programs. Besides, it will keep reporting a growing body of data to WHO GLASS allowing further comparison with international studies.

## Data Availability Statement

The raw data supporting the conclusions of this article will be made available by the authors, without undue reservation.

## Ethics Statement

The studies involving human participants were reviewed and approved by Comitê de Ética em Pesquisa Pontifícia Universidade Católica do Paraná PUCPR. Written informed consent from the participants or their legal guardians/next of kin was not required to participate in this study in accordance with the national legislation and the institutional requirements.

## Author Contributions

MP and GSA wrote the manuscript and conducted the statistical analysis. MO, BA, KN, AD, and VD collected the data. RB and FR compiled the data and provided the dashboard. RJ and AA reviewed the data. All authors reviewed and contributed with inputs to the manuscript.

## Conflict of Interest

The authors declare that the research was conducted in the absence of any commercial or financial relationships that could be construed as a potential conflict of interest.

## Supplementary Material

Supplementary Figure 1 Questionnaire submitted to the Sentinels candidates to participate in BR-GLASS.

Supplementary Figure 2 Description of the data submitted to the BR-GLASS by the hospitals.

Supplementary Figure 3 Additional Dashboard data from the BR-GLASS Program.

Supplementary Table 1 BR-GLASS Resistance Rates compared with international data stratified by country.

Supplementary Table 2 Antibiogram Test Results BR-GLASS.
