# Supplementary material for: The Experience of Implementing a National Antimicrobial Resistance Surveillance System in Brazil
Source: Front Public Health. 2021 Jan 14;8:575536. doi: 10.3389/fpubh.2020.575536 (PMC7841397; doi:10.3389/fpubh.2020.575536)
Supplement: Supplementary Figure 1 — Questionnaire submitted to the Sentinels candidates to participate in BR-GLASS. [file Data_Sheet_1.DOCX]

# Supplemental Figure 01

**Questionário para os serviços candidatos à Rede Sentinela BR-GLASS**

**Dados Cadastrais:**

Nome da Instituição:________________________________________________

CNPJ: ___________________________________________________________

Endereço: ________________________________________________________

Cidade: __________________________________ Estado:_________________

N. de Leitos: N. Leitos UTI: N. Leitos UTI-Neonatal:_________

N. de Internações por ano: ___________________________________________

**Questionário Hospital:**

1. O Hospital possui atendimento ambulatorial e hospitalar?

☐ Sim ☐ Não

1. O Hospital possui Núcleo Hospitalar de Epidemiologia ativo e de acordo com a Portaria 2254/2010 do Ministério da Saúde?

☐ Sim ☐ Não

1. O Hospital possui Licença Sanitária atualizada?

☐ Sim ☐ Não

1. A instituição participa da Rede Sentinela da ANVISA?

☐ Sim ☐ Não

1. Possui laboratório de Microbiologia próprio?

☐ Sim ☐ Não

1. O Hospital possui Serviço de Controle de Infecções Relacionadas à Assistência em Saúde - SCIRAS?

☐ Sim ☐ Não

1. O hospital notifica mensalmente quantos agravos em saúde, previstos na Portaria 204/2016/MS de Notificação Compulsória de Doenças?

☐ até 10 ☐ de 11a 20 ☐ de 21 a 50 ☐Acima de 50

1. O hospital possui em seu corpo clínico médico infectologista?

☐ Sim ☐ Não Se afirmativo quantos?________

1. O hospital possui em seu corpo clínico médico epidemiologista?

☐ Sim ☐ Não Se afirmativo quantos?________

1. O hospital é acreditado por organização nacional ou internacional?

☐ Sim ☐ Não

1. Se afirmativo, por qual organização?

☐ISQua ☐ JCAHO ☐ ONA ☐Outra:______________

**Questionário Laboratório:**

1. O Laboratório possui Sistema de Gestão da Qualidade implantado?

☐ Sim ☐ Não

1. Se afirmativo, qual norma segue?

☐ ISO 15189 ☐ ISO 17025 ☐ RDC 33 ☐Outra:______________

1. O laboratório é acreditado por organização nacional ou internacional?

☐ Sim ☐ Não

1. Se afirmativo, por qual organização?

☐CAP ☐ DICQ ☐ ONA ☐PALC ☐ Outra:______________

1. Tem na equipe do laboratório profissional especialista em Microbiologia?

☐ Sim ☐ Não

1. Utiliza qual critério de interpretação dos Testes de Sensibilidade a Antimicrobianos

☐ BrCAST ☐EUCAST ☐CLSI ☐Outro___________________

1. Dispõe de quais métodos para realizar o teste de sensibilidade a antibióticos – TSA (anotar todos implantados)?

☐difusão em disco ☐ microdiluição em caldo ☐diluição em ágar

☐gradiente de concentração ☐ macrodiluição em caldo

1. Realiza controle de qualidade do TSA?

☐ Sim ☐ Não

1. Se afirmativo, com qual frequência?

☐Mensalmente ☐ Semanalmente ☐Diariamente

1. Quais cepas utiliza para realizar o controle de qualidade do TSA?

☐*E. coli* ATCC 25922 ☐ *S. aureus* ATCC 25923

☐ *P. aeruginosa* ATCC 27853 ☐ *E. faecalis* ATCC 29212

☐ *S. pneumoniae* ATCC 49619 ☐ *H. influenzae* ATCC 49766

☐ Outros (relatar todos) _______________________

__________________________ _______________________

__________________________ _______________________

1. Realiza testes fenotípicos para detecção de mecanismos de resistência?

☐ Sim ☐ Não

1. Se afirmativo, assinale quais:

☐ácido fenilborônico ☐EDTA ☐Cloxacilina

☐ácido dipicolínico ☐Hodge ☐CarbaNP

☐Blue-Carba ☐Polimixina NP ☐Outros (relatar todos)

__________________________ _______________________

__________________________ _______________________

1. Realiza controle de qualidade de detecção de mecanismos de resistência?

☐ Sim ☐ Não

1. Se afirmativo, com qual frequência?

☐Mensalmente ☐ Semanalmente ☐Diariamente

1. Quais cepas utiliza para realizar o controle de qualidade de detecção de mecanismos de resistência?

☐ *K. pneumoniae* ATCC 700603 ☐ *S. aureus* NCTC 12493

☐ *E. faecalis* ATCC 51299 ☐ *H. influenzae* ATCC 49247

☐ Outras (relatar todas)

__________________________ _______________________

__________________________ _______________________

1. Realiza detecção molecular de mecanismos de resistência?

☐ Sim ☐ Não

1. Se afirmativo, quais genes pesquisa de rotina?

☐ *bla*_KPC_ ☐ *bla*_NDM_ ☐ *bla*_OXA-48_ ☐ *bla*_OXA-23_

☐ *bla*_SPM_ ☐ *bla*_IMP_ ☐ *bla*_VIM_ ☐ *mcr-1*

☐ *vanA* ☐ *vanB* ☐ *bla*_CTX-M_ ☐ *bla*_SHV_

☐ Outros (relatar todos)

1. Qual o número total de culturas microbiológicas processadas mensalmente?

___________________________________________________________

1. Qual o número total de hemoculturas positivas mensalmente?

___________________________________________________________

1. Qual o número total de uroculturas positivas mensalmente?

___________________________________________________________

1. Qual o número total de culturas cervicais/genitais positivas para *Neisseria gonorrhoeae*, mensalmente?

___________________________________________________________

# ANEXO II

# Sistema de Pontuação dos Questionários

**TABELAS DE PONTUAÇÃO**

| **QUESTÕES HOSPITAL** | |
| --- | --- |
| NÚMERO | PONTOS |
| 1 | 3 |
| 2 | 5 |
| 3 | 3 |
| 4 | 5 |
| 5 | 5 |
| 6 | 4 |
| 7 | até 10: 1  11-20: 2  21-50: 3  > 50: 5 |
| 8 | 2 pontos a cada profissional |
| 9 | 2 pontos a cada profissional |
| 10 | 5 |
| 11 | NA |

NA – Não se aplica.

| **QUESTÕES LABORATÓRIO** | |
| --- | --- |
| NÚMERO | PONTOS |
| 1 | 5 |
| 2 | NA |
| 3 | 5 |
| 4 | NA |
| 5 | 5 |
| 6 | NA |
| 7 | 2 pontos a cada método |
| 8 | 5 |
| 9 | NA |
| 10 | 01 ponto por cepa |
| 11 | NA |
| 12 | 01 ponto por teste |
| 13 | 5 |
| 14 | NA |
| 15 | 01 ponto por cepa |
| 16 | 3 |
| 17 | 01 ponto por teste |
| 18 | NA |
| 19 | NA |
| 20 | NA |
| 21 | NA |

NA – Não se aplica.
