# Supplementary material for: The Experience of Implementing a National Antimicrobial Resistance Surveillance System in Brazil
Source: Front Public Health. 2021 Jan 14;8:575536. doi: 10.3389/fpubh.2020.575536 (PMC7841397; doi:10.3389/fpubh.2020.575536)
Supplement: Supplementary Figure 2 — Description of the data submitted to the BR-GLASS by the hospitals. [file Data_Sheet_2.DOC]

**Layout - BRGLASS**

| **N°** | **Campo** | **O** | **Tipo** | **Tamanho / Formato** | **Descrição** |
| --- | --- | --- | --- | --- | --- |
| **1** | id_estabelecimento | **X** | Integer |  | Código do Estabelecimento que solicitou o exame. |
| **2** | co_matriz | **X** | Varchar | 2 | Matriz da amostra. Os valores são:  HU - Humano  AN - Animal  AM - Ambiental  PR - Produto |
| **3** | id_estabelecimento_executor | **X** | Integer |  | Código do Estabelecimento que executou o exame. |
| **4** | id_material | **X** | Integer |  | Código do Material Biológico – v[ide tabela de Material Biológico](#Tabela_Material_Biologico)**.** |
| **5** | co_amostra | **X** | Varchar | 12 | Código de Identificação da amostra no sistema de origem. |
| **6** | nu_amostra | **X** | Varchar | 1 | Número da Amostra. Os valores são:  U – Amostra Única  1 – 1ª Amostra  2 – 2ª Amostra  3 – 3 ª Amostra |
| **7** | dt_coleta | **X** | Date | 10  dd/mm/yyyy | Data da Coleta. |
| **8** | ds_motivo |  | Varchar | 255 | Descrição do Motivo. |
| **9** | dt_cadastro | **X** | Date | 10  dd/mm/yyyy | Data do Cadastro da Amostra. |
| **10** | id_paciente | **X** | Varchar | 12 | Número único de identificação do paciente no sistema de origem. |
| **11** | cns_paciente |  | Varchar | 15 | Cartão Nacional de Saúde do Paciente. |
| **12** | no_paciente | **X** | **Varchar** | 100 | Nome completo do paciente. |
| **13** | no_mae |  | Varchar | 100 | Nome da mãe do paciente. |
| **14** | dt_nascimento |  | Date | 10  dd/mm/yyyy | Data de nascimento do paciente. |
| **15** | nu_idade | **X** | Integer |  | Idade do paciente. |
| **16** | tp_idade | **X** | Varchar | 1 | Tipo da idade. Os valores são:  1 - Hora(s)  2 - Dia(s)  3 - Mês(es)  4 - Ano(s) |
| **17** | co_genero | **X** | Varchar | 1 | Gênero do paciente. Os valores são:  M - Masculino  F – Feminino  I - Ignorado |
| **18** | co_municipio | **X** | Varchar | 5 | Código do IBGE do Município de residência do paciente. |
| **19** | co_atendimento | **X** | Varchar | 1 | Tipo de atendimento. Os valores são:  A - Ambulatorial  H - Hospitalar |
| **20** | dt_internacao |  | Date | 10  dd/mm/yyyy | Data da internação do paciente. |
| **21** | co_origem |  | Integer |  | Local de origem do paciente. Os valores são:  1 - Comunitário  2 – Hospitalar  3 - Ignorado |
| **22** | co_unidade_origem |  | Integer |  | Departamento da unidade de origem do paciente. Os valores são:  1 - UTI Adulto  2 - UTI Pediátrica  3 - UTI-Neonatal  4 - UTI-Neonatal/Pediátrica  5 - Semi-Intensiva  6 – Pronto Socorro  7 – Pronto Atendimento  8 – Unidade de Internação  9 - Ignorado |
| **23** | ds_unidade_origem |  | Varchar |  | Descrição da unidade de origem do paciente. |
| **24** | pac_desfecho |  | Varchar |  | Desfecho da internação do paciente. Os valores são:  1 - Alta  2 - Transferência  3 - Óbito  4 - Internado  5 - Ignorado |
| **25** | id_microrganismo | **X** | Integer |  | Código do Microrganismo analisado – vide tabela de Microrganismos. |
| **26** | co_perfil | **X** | Integer |  | Código do perfil do microrganismo. O valor padrão é 1. Caso o microrganismo tenha sido testado novamente na mesma amostra/exame o valor do perfil é incrementado um inteiro. Ex: S. aureus → 2, S.aureus → 3... |
| **27** | rs_sorotipo |  | Varchar | 255 | Resultado do teste de sorotipagem. |
| **28** | rs_beta_lactamase |  | Varchar | 255 | Resultado do teste de Beta-lactamase. |
| **29** | rs_esbl |  | Varchar | 255 | Resultado do teste de ESBL. |
| **30** | rs_carbapenemase |  | Varchar | 255 | Resultado do teste de Carbapenemase. |
| **31** | rs_mrsa |  | Varchar | 255 | Resultado do teste de MRSA screening test. |
| **32** | rs_icr |  | Varchar | 255 | Resultado do teste de ICR. |
| **33** | dt_liberacao | **X** | Date | 10  dd/mm/yyyy | Data da liberação do resultado. |
| **34** | id_antibiotico | **X** | Integer |  | Código do Antibiótico testado – vide tabela de Antibióticos. |
| **35** | id_criterio_tsa | **X** | Integer |  | Identificação do critério usado no teste de sensibilidade. – vide tabela de Critério de TSA. |
| **36** | id_metodo_tsa | **X** | Integer |  | Método usado no teste de sensibilidade. – vide tabela de Método de TSA. |
| **37** | id_resultado | **X** | Integer |  | Resultado do teste de sensibilidade. – vide tabela de Resultados. |
| **38** | valor |  | Float |  | Valor do halo do resultado do teste de sensibilidade. |

**Regras**:

Os campos marcados com “**X”** na coluna **O** são obrigatórios.

Os campos **20**, **21**, **22** e **23** se tornam obrigatórios quando o **campo 19** for preenchido com a opção “**HOSPITALAR**”.

Se o campo **19** for preenchido com a opção “**AMBULATORIAL**” o campo **22** deve ser preenchido com uma das opções: **6**, **7** ou **9**.

**Informações Complementares****:**

No total de uroculturas positivas e negativas.

No total de hemoculturas positivas e negativas.

No total de coproculturas positivas e negativas.

No total de secreções genitais positivas e negativas.
