# Supplementary material for: The Experience of Implementing a National Antimicrobial Resistance Surveillance System in Brazil
Source: Front Public Health. 2021 Jan 14;8:575536. doi: 10.3389/fpubh.2020.575536 (PMC7841397; doi:10.3389/fpubh.2020.575536)
Supplement: Supplementary Figure 3 — Additional Dashboard data from the BR-GLASS Program. [file Data_Sheet_3.DOCX]

**Supplemental Figure 03:** Additional Dashboard data from the BR-GLASS Program

**
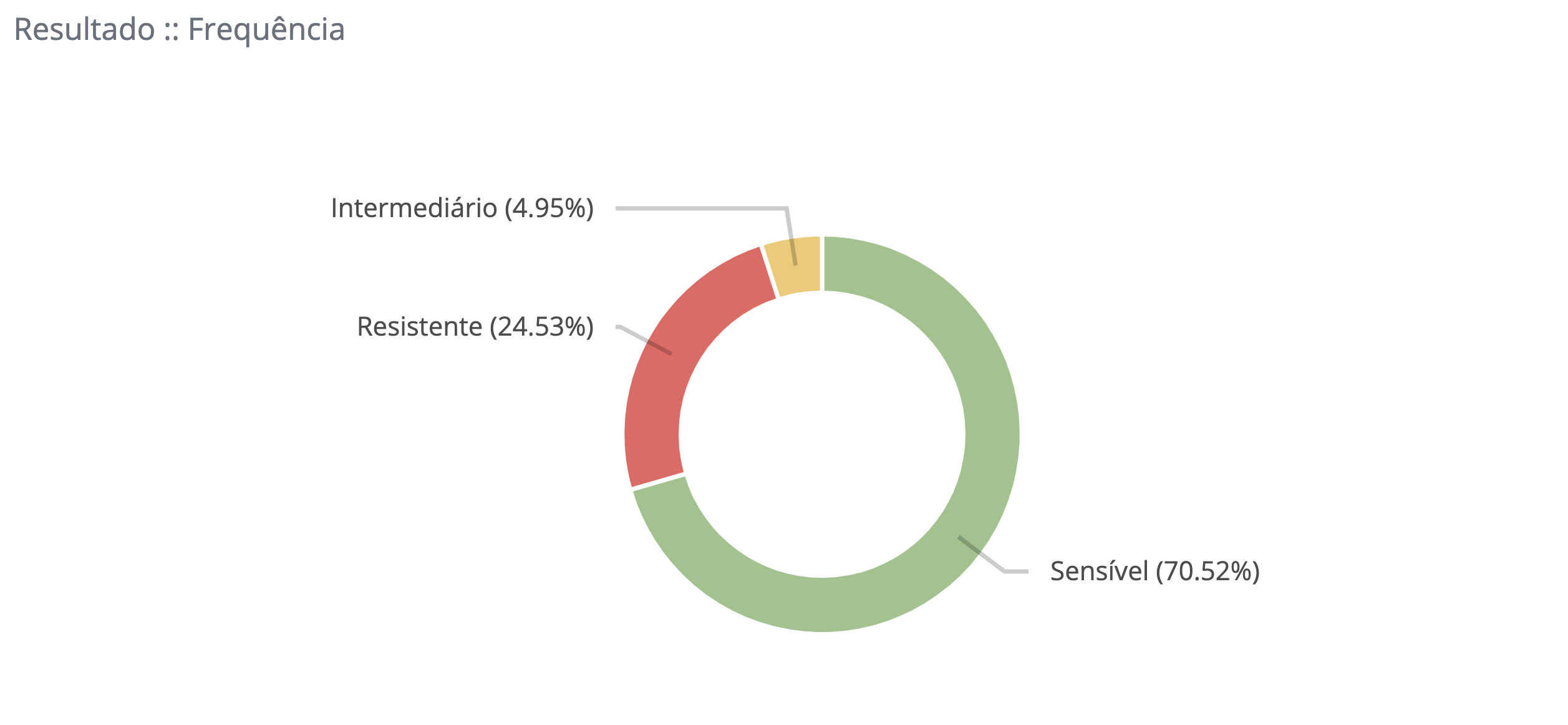
**

1. Frequency of the overall results (Susceptible, Intermediate, Resistant)


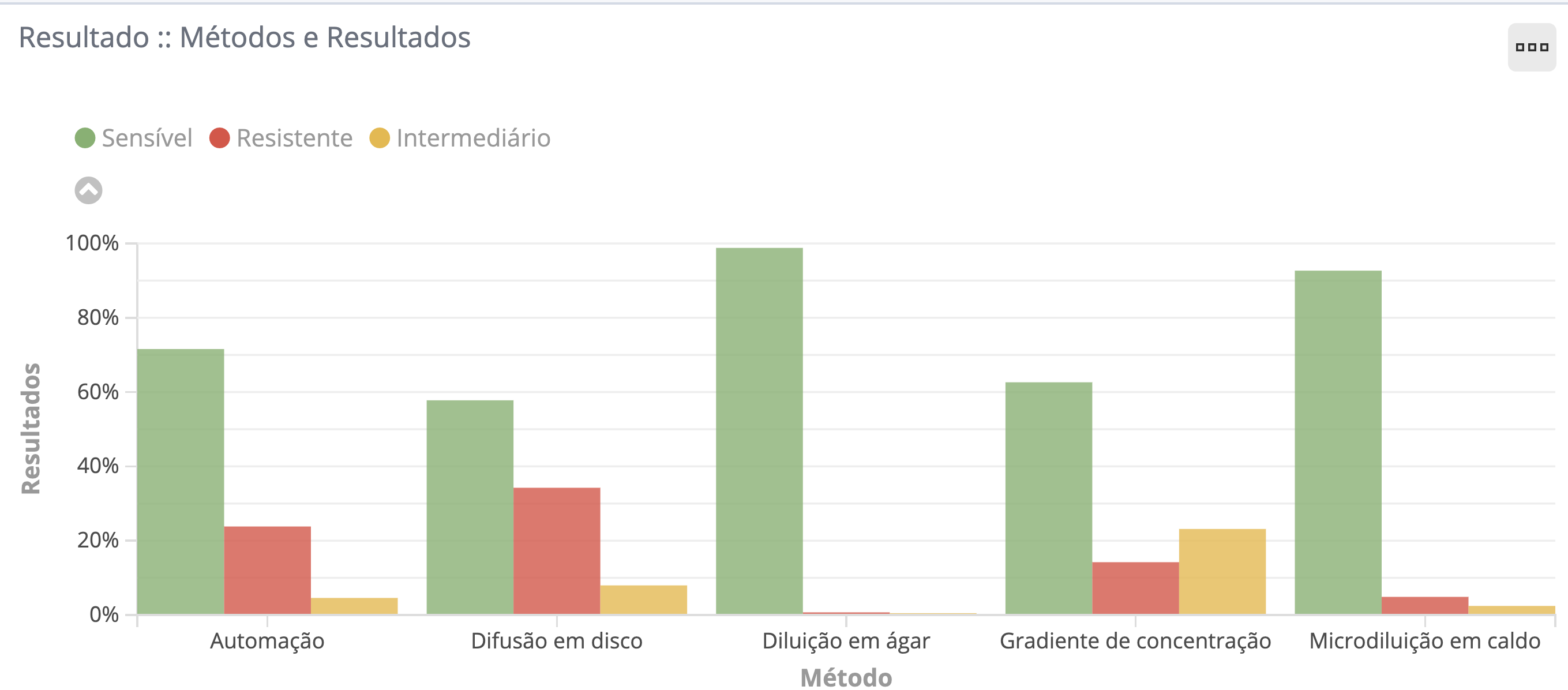


1. Distribution of susceptible, intermediate and resistant strains by method used for AST

**
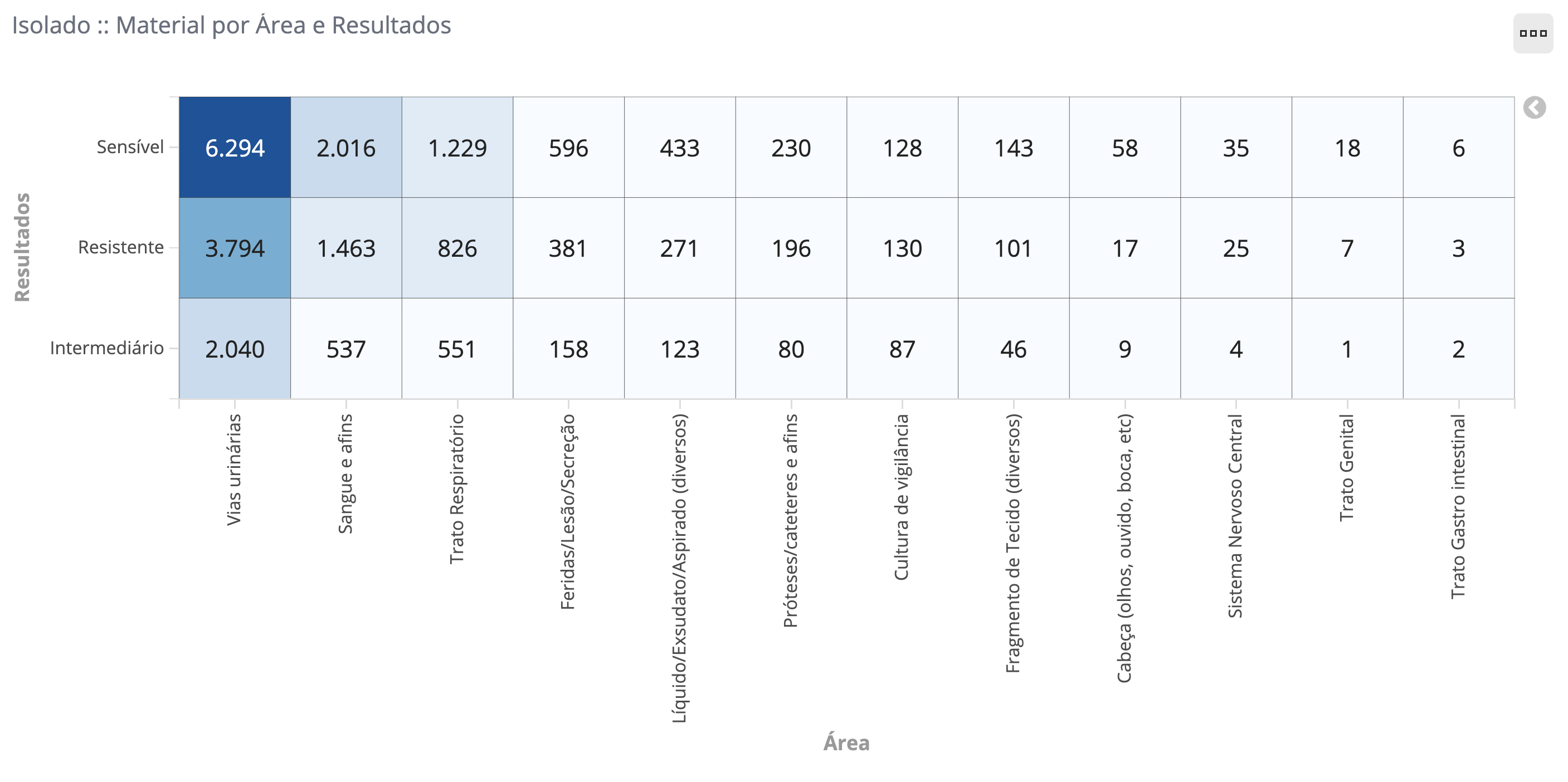
**

1. Total Number of isolates by anatomical site, distributed through categories (susceptible, intermediate and resistant)


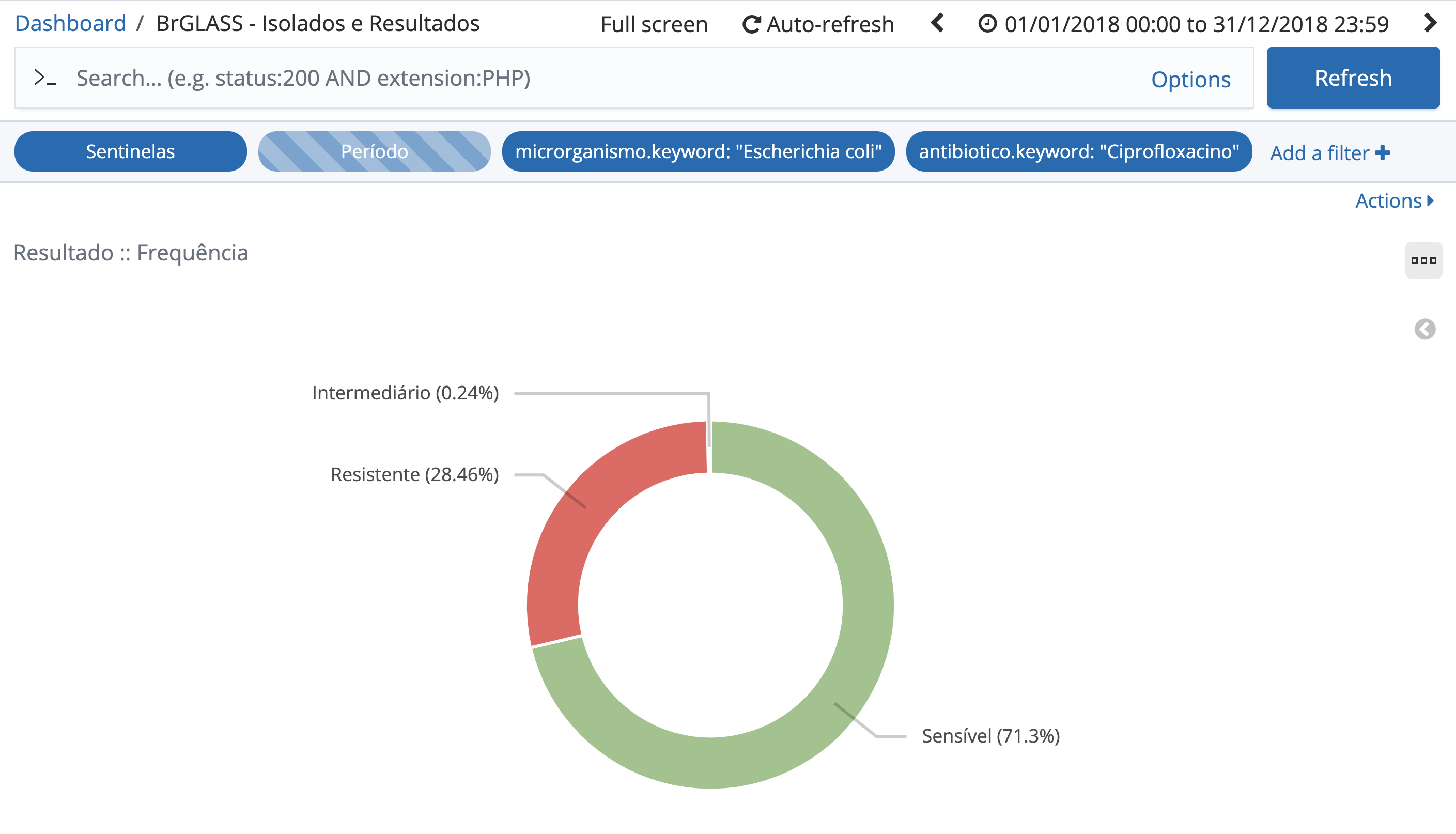


1. Example of filter use by BR-GLASS, showing the percentage of resistant and susceptible strains of *E.coli*, regarding ciprofloxacin


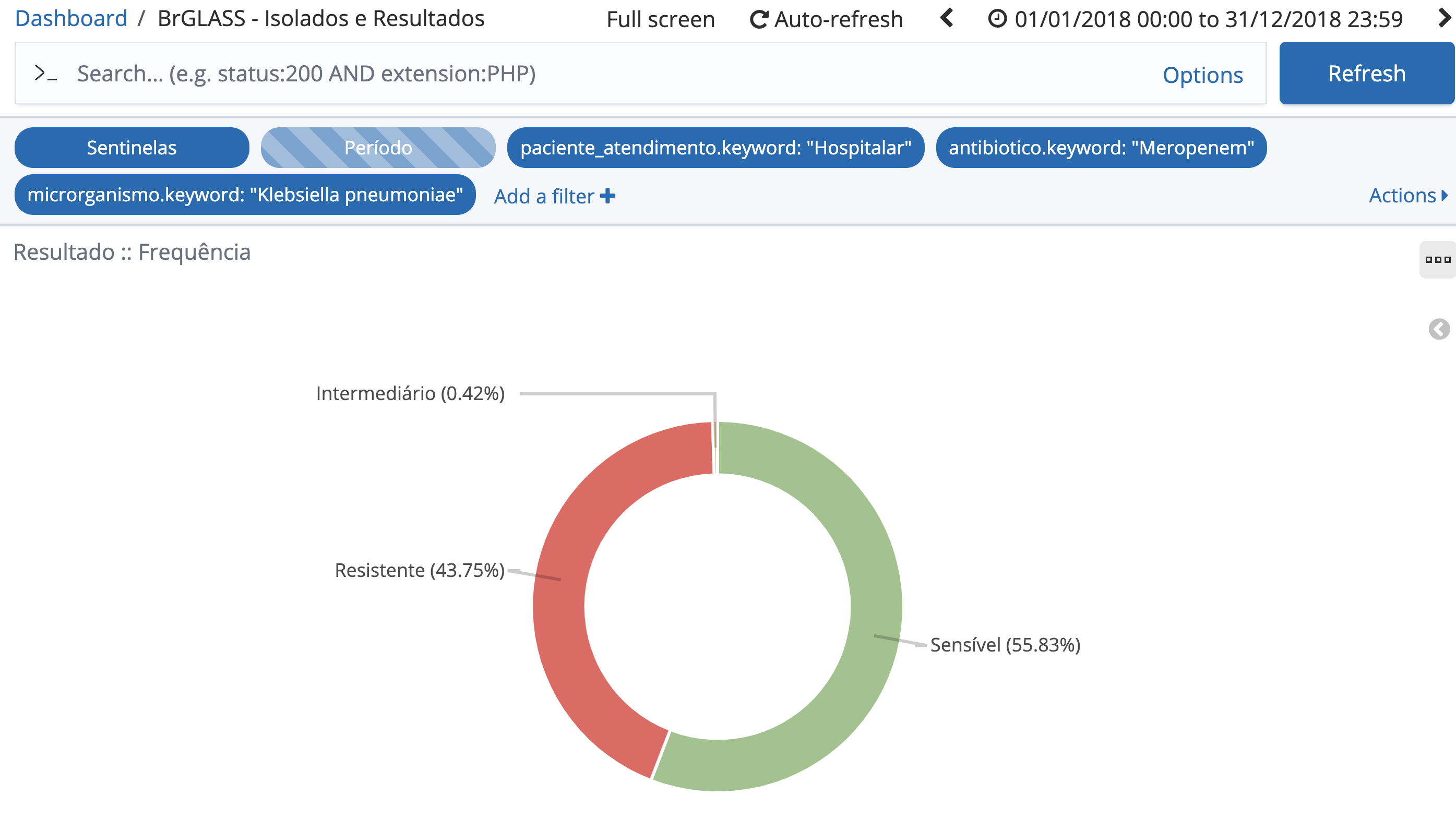


1. Another example, showing the high percentage of resistant strains of *K. pneumoniae*, in hospital isolates regarding meropenem.
